# In Situ Formation of Fibronectin‐Enriched Protein Corona on Epigenetic Nanocarrier for Enhanced Synthetic Lethal Therapy

**DOI:** 10.1002/advs.202307940

**Published:** 2024-03-14

**Authors:** Zhangyi Luo, Zhuoya Wan, Pengfei Ren, Bei Zhang, Yixian Huang, Raymond E. West, Haozhe Huang, Yuang Chen, Thomas D. Nolin, Wen Xie, Junmei Wang, Song Li, Jingjing Sun

**Affiliations:** ^1^ Center for Pharmacogenetics Department of Pharmaceutical Science University of Pittsburgh School of Pharmacy Pittsburgh PA 15213 USA; ^2^ Department of Pharmacy and Therapeutics University of Pittsburgh School of Pharmacy Pittsburgh PA 15213 USA; ^3^ Department of Pharmaceutical Sciences and Computational Chemical Genomics Screening Center University of Pittsburgh School of Pharmacy Pittsburgh PA 15213 USA; ^4^ Department of Pharmaceutical Sciences College of Pharmacy University of Nebraska Medical Center Omaha NE 68106 USA; ^5^ Fred & Pamela Buffett Cancer Center University of Nebraska Medical Center Omaha NE 68106 USA

**Keywords:** immunotherapy, nanocarrier, protein corona, synthetic lethal therapy, ultra‐Small

## Abstract

PARP inhibitors (PARPi)‐based synthetic lethal therapy demonstrates limited efficacy for most cancer types that are homologous recombination (HR) proficient. To potentiate the PARPi application, a nanocarrier based on 5‐azacytidine (AZA)‐conjugated polymer (PAZA) for the codelivery of AZA and a PARP inhibitor, BMN673 (BMN) is developed. AZA conjugation significantly decreased the nanoparticle (NP) size and increased BMN loading. Molecular dynamics simulation and experimental validations shed mechanistic insights into the self‐assembly of effective NPs. The small PAZA NPs demonstrated higher efficiency of tumor targeting and penetration than larger NPs, which is mediated by a new mechanism of active targeting that involves the recruitment of fibronectin from serum proteins following systemic administration of PAZA NPs. Furthermore, it is found that PAZA carrier sensitize the HR‐proficient nonsmall cell lung cancer (NSCLC) to BMN, a combination therapy that is more effective at a lower AZA/BMN dosage. To investigate the underlying mechanism, the tumor immune microenvironment and various gene expressions by RNAseq are explored. Moreover, the BMN/PAZA combination increased the immunogenicity and synergized with PD‐1 antibody in improving the overall therapeutic effect in an orthotopic model of lung cancer (LLC).

## Graphical abstract

An ultrasmall nanocarrier for the co‐delivery of azacytidine (AZA) and BMN673 is developed. The nanocarrier is capable of recruiting fibronectin (FN) enriched protein corona, resulting in effective tumor targeting and penetration through FN/ITGA5 mediated transcytosis. Co‐delivery of AZA and BMN673 to tumors enhances the synthetic lethal therapy and elicits antitumor immune response through the epigenetic modulation of AZA. The figure is created with BioRender.com.

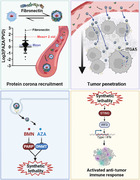

## 1 Introduction

Inhibition of PARP1 is a clinically effective synthetic lethal therapy in homologous recombination (HR)‐deficient cancers by generating double‐strand breaks (DSBs).^[^ ^1^ ^]^ However, it offers insubstantial benefits for the majority of cancer types that are HR‐proficient, such as nonsmall cell lung cancer (NSCLC). Moreover, cancer patients succumb to PARPi resistance through various feedback mechanisms such as induction of the anomalous hypermethylation of tumor suppressor gene promoters.^[^ ^2^ ^]^ A key player in the resistance is DNA methyltransferase 1 (DNMT1), which is overexpressed in tumors, mediating abnormal DNA methylation patterns that epigenetically silence the expression of tumor suppressor genes.^[^ ^3^ ^]^ Intriguingly, epigenetic modifiers like DNMT1 inhibitors (DNMTi) interfere with the process of DNA repair, rendering HR‐proficient tumor cells of HR‐deficient phenotype and sensitizing them to PARPi and other DNA‐damaging agents.^[^ ^4^ ^]^ From the immunological perspective, DNA‐damaging agents have the potential to convert immunologically “cold” tumors to “hot,” augmenting immunotherapy via mechanisms like cyclic GMP‐AMP synthase (cGAS)‐stimulator of interferon genes (STING) pathway,^[^ ^5^ ^]^ elevation of tumor mutation burden,^[^ ^6^ ^]^ and the upregulated generation of HLA‐neoantigen complex.^[^ ^7^ ^]^ Meanwhile, DNMTi could upregulate the expression of the MHC I gene,^[^ ^8^ ^]^ enhancing the immune system's recognition of tumor cells through increased presentation of neoantigens. Taken together, a combination of DNMTi with PARPi represents an attractive approach to improve the treatment through several synergistic mechanisms.

However, oral administration of PARPi shows low bioavailability and poor drug accumulation in the tumor tissue, necessitating the use of high doses in patients, which may cause off‐target toxicity and limit its long‐term use.^[^ ^2b^ ^]^ Similarly, DNMTi such as 5‐azacytidine (AZA) and decitabine are unstable and show a limited duration of action due to enzymatic inactivation by cytidine deaminase (CDA).^[^ ^9^ ^]^ Nanocarriers have emerged as a powerful tool in enhancing the bioavailability of free drugs.^[^ ^10^ ^]^ However, it is still challenging to effectively co‐load water‐insoluble PARPi and water‐soluble AZA into a single nanocarrier. In addition, tumor targeting and penetration represent another major barrier for nanoparticles (NPs)‐mediated cancer therapy. Smaller NPs often exhibit superior tumor accumulation and penetration.^[^ ^11^ ^]^ It is generally assumed that smaller NPs shall be more effective in passing through paracellular space in both tumor endothelium lining as well as the tumor cells and stromal cells following extravasation.^[^ ^12^ ^]^ However, a recent study utilizing Au NPs of varying sizes has revealed that most NPs enter tumors through active transcytosis processes within endothelial cells.^[^ ^13^ ^]^ Contrary to previous assumptions, passive diffusion through leaky vasculature seems to be a secondary route of entry even for 15 nm Au NPs. Nonetheless, the exact mechanisms governing this trans‐endothelial process have remained elusive, particularly for the more effective smaller NPs. In addition, it remains uncertain whether this phenomenon extends to other nanocarrier systems, such as polymeric NPs. Moreover, although increasing evidence demonstrates that smaller NPs achieve deeper tumor penetration, the underlying mechanism is still underexplored. Unraveling these puzzles holds the key to engineering the next generation of nanocarriers, ensuring deeper tumor penetration and potentially revolutionizing therapeutic outcomes.

In this work, we developed an ultrasmall nanocarrier PAZA for co‐delivery of DNMTi AZA and PARPi BMN673 to enhance synthetic lethal therapy in HR‐proficient tumors. By conjugating AZA to a PVD polymeric backbone, the particle size was significantly reduced to 12 nm. This reduction facilitated tumor penetration through clathrin‐mediated transcytosis, a departure from the generally assumed paracellular transport mechanism. Through proteomics and genetic knocking out (KO) approaches, we found that fibronectin was enriched in the protein corona (PC) surrounding PAZA nanocarrier, which interacted with the ITGA5 receptor on tumor cells, contributing to effective tumor uptake and penetration. The in situ formed PC‐PAZA NPs loaded with BMN673 exhibited remarkable antitumor activity in HR‐proficient NSCLC at low dosages. Furthermore, BMN/PAZA increased the immunogenicity, synergizing effectively with PD‐1 antibodies to combat orthotopic LLC lung cancer.

### 1.1 Preparation and Characterization of Ultra‐Small Micelles

**Figure 1:**
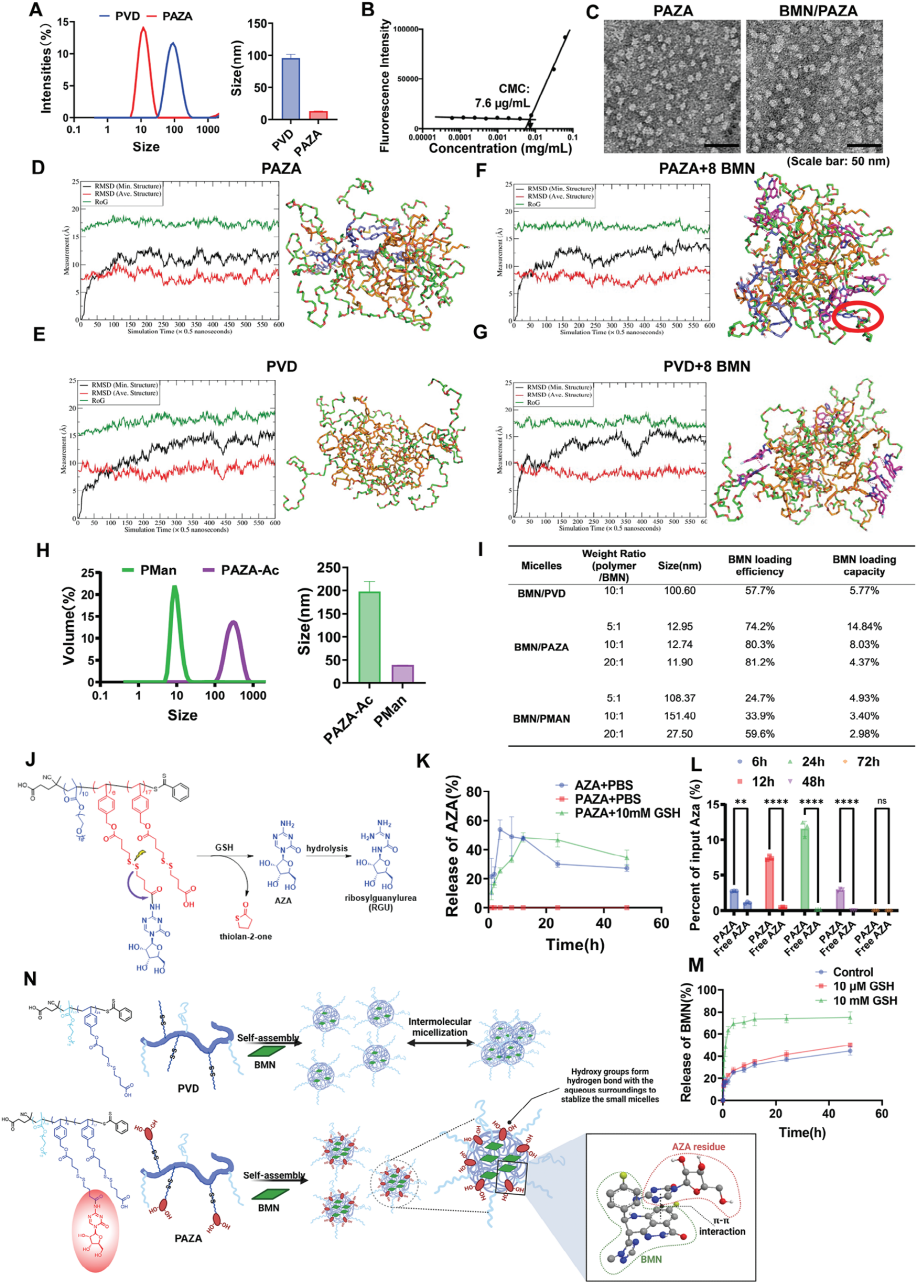
Characterization and modeling of PAZA polymer. A) Size of PVD and PAZA micelles. B) Critical micelle concentration of PAZA polymer. C) Morphology of PAZA and BMN loaded PAZA by TEM. (Magnification: 62kx, bar = 50 nm) D–G) Time courses of root‐mean‐square deviations (RMSD) of heavy atoms and radius of gyration (RoG) and representative conformations of D) PAZA; E) PVD; F) PAZA+8 BMN molecules; G) PVD + 8 BMN molecules. The following color schemes were applied: greenish sticks for hydrophobic parts of the residues, brownish sticks for hydrophilic parts of the residues, and magenta sticks for the AZA substructure. Red cycle: π–π interactions moiety between PAZA and BMN. H) Size of acetylated‐PAZA (PAZA‐Ac) and PMan micelle. I) Drug loading parameters and size of BMN loaded PVD, PAZA, and PMan. J) Scheme of the self‐immolation release of AZA from PAZA under redox conditions. K) Release of AZA from PAZA under redox conditions. L) Intracellular release of AZA from PAZA detected by LC/MS. (*n* = 3, ^*^ *p* < 0.05, ^**^ *p* < 0.01, ^***^ *p* < 0.001, ^****^ *p* < 0.0001). M) In vitro release of BMN from PAZA under different GSH concentrations. N) Illustration of the self‐assemble and drug loading mechanism of PVD and PAZA. The figure is created with BioRender.com.

The PVD polymer backbone was synthesized by reversible addition‐fragmentation chain transfer (RAFT) polymerization, and the AZA‐conjugated polymer (PAZA) was obtained by conjugation of AZA to PVD backbone (Figure S1, Supporting Information). The structures were characterized by NMR and IR spectra (Figures S2 and S3, Supporting Information). PVD could form NPs with an average diameter of 100 nm in aqueous solution. Interestingly, conjugation of six units of AZA to PVD drastically decreased the NP size to 12.3 nm (**Figure** **1**A). PAZA micelles showed a low critical micelle concentration (CMC) value of 0.0076 mg mL^−1^ (Figure 1B), indicating a likely excellent micelle stability upon dilution. The PAZA carrier was able to load BMN into spherical NPs with a drug loading capacity as high as 14.8%, which was much higher than that of PVD carrier (≈5.8%) (Figure 1C,I). In addition, the formulations showed excellent stability both at room temperature and through the lyophilization process (Figure S4, Supporting Information).

It has been reported that reducing the particle size of polymeric micelles often compromises the encapsulation capacity and stability of drug‐loaded micelles.^[^ ^14^ ^]^ Intriguingly, we found that conjugation of AZA markedly reduced the nanocarrier size to 12 nm, while simultaneously enhancing drug loading efficiency. To deliver into the mechanism of interaction between the BMN molecule and PVD or PAZA polymer, we conducted molecular dynamics (MD) simulations (Figure S5, Supporting Information). The model system comprised one polymer copy and eight BMN copies, with solvent effects simulated through explicit water MD and a subsequent Generalized Born surface area (GBSA) model. After a series of MD simulations, micelle structures were formed for both PAZA (Figure 1D) and PVD polymers (Figure 1E). It was shown that the conjugation of AZA induced a reconfiguration of the polymer segments, facilitating the formation of more stable micelles with a tighter packing of the hydrophobic core (brownish sticks) compared to PVD. In swarm MD simulations, BMN formed similar hydrogen bonding with both PVD and PAZA with 2.32 and 2.36 hydrogen‐bonds on average, respectively. However, BMN showed stronger π–π interactions with PAZA than with PVD, which comes from pyrimidine ring of AZA (Figure 1F,G).

Molecular Mechanics/Poisson–Boltzmann Surface Area – Water Solvent Accessible Surface (MM‐PBSA‐WSAS) method was used to estimate the free energy of binding between BMN and the nanocarriers, providing insights into the thermodynamics of drug encapsulation within the nanocarriers. We first calculated MM‐PBSA‐WSAS free energies for BMN itself, PAZA or PVD polymer itself, and PAZA + 8 BMN and PVD + 8 BMN. Then we calculated the free energy change upon BMN binding, Δ *G*_binding_ = *G*_complex_  − (*G*_polyer_ + 8 × *G*_BMN_) (Table S1, Supporting Information). The calculated Δ*G*_binding_ values were −44.43 and −38.16 kcal mol^−1^ for PAZA and PVD, respectively. This result explained why PAZA has a better loading capacity than PVD. Moreover, we calculated the root‐mean‐square deviation (RMSD) of the heavy atoms to describe the dynamics of the polymer and polymer‐drug complex. PAZA/BMN complex exhibited higher stability than the PVD/BMN complex, as evidenced by the smaller fluctuation in the RMSD values for the former (black curves of Figure 1F,G).

Figure 1N shows the model we proposed to elucidate the improved performance of PAZA. The formation of large micelles by the PVD polymer can be attributed to the mechanism of multi‐micelle aggregates, wherein the initially formed small micelles exhibit limited stability, resulting in rapid flocculation and the subsequent formation of larger multi‐micelle aggregates.^[^ ^15^ ^]^ In contrast, for PAZA, the hydroxyl groups of AZA molecules at the interface can engage in hydrogen bonding with the aqueous surroundings, thereby stabilizing the initially formed small micelles. In addition, the π‐π interaction between the pyrimidine ring of AZA and BMN (Figure 1F,G) helps to improve both BMN loading capacity and the formulation stability. To further test our model, we synthesized the acetalized PAZA (PAZA‐ac) (Figure S6, Supporting Information) to eliminate the effect of hydrogen bond of PAZA. We also synthesized mannitol‐conjugated PVD (PMan) (Figure S7, Supporting Information) to attenuate the π–π interaction between the pyrimidine ring of AZA and BMN. As shown in Figure 1H, PAZA‐ac showed drastically increased size, but PMan was able to maintain the ultra‐small size, suggesting that the hydroxyl group of AZA played an important role in forming the ultrasmall micelle. In addition, PMan suffered a significant drop in drug loading capacity compared to PAZA (Figure 1I), suggesting that the pyrimidine ring of AZA contributed significantly to BMN loading through π–π interaction (Figure 1N). This information might be of value in the future design of other ultrasmall‐sized drug formulations.

We incubated AZA or PAZA with tumor lysate for 24 and 48 h, respectively. The free AZA group showed a very low concentration of AZA after 24 h. In contrast, the PAZA group maintained a significantly higher concentration of AZA in the tumor lysate after both 24 and 48 h. This result indicated that in addition to improved performance in loading a drug, PAZA can protect AZA from hydrolysis and enzymatic degradation such as by cytidine deaminase ^[^ ^16^ ^]^ (Figure S8, Supporting Information). PAZA is a redox‐responsive self‐immolation carrier that can release the parent AZA due to the formation of thiolan‐2‐one (Figure 1J; Figure S9, Supporting Information). The release study showed that no AZA was released from PAZA in the PBS (pH 7.4) without GSH, but the fast release of AZA was observed in the presence of 10 mM GSH (Figure 1K). The accumulative amount of released AZA was decreased after 12 h likely due to that the released AZA was not stable in an aqueous solution and gradually hydrolyzed to ribosylguanylurea ^[^ ^17^ ^]^ (Figure 1J). Moreover, in contrast to the rapid intracellular degradation observed with free AZA treatment, PAZA demonstrated a sustained release of AZA, maintaining significantly higher intracellular drug concentrations for 48 h. This further underscores the excellent protective effect of PAZA nanocarrier in AZA delivery (Figure 1L). We also assessed the release kinetics of BMN from the BMN/PAZA formulation. In its absence or low GSH environment, only less than 25% of BMN loaded in PAZA was released in 2 h, and the slow kinetics of release was extended for 48 h. However, upon exposure to 10 mm GSH, the release of BMN from the PAZA micelles was greatly accelerated and more than 60% BMN was released. The accelerated drug release is likely due to the disassembly of the micelles, which was induced by the cleavage of the disulfide link (Figure 1M).

### 1.2 Efficient Tumor Penetration via Clathrin‐Mediated Transcytosis

**Figure 2:**
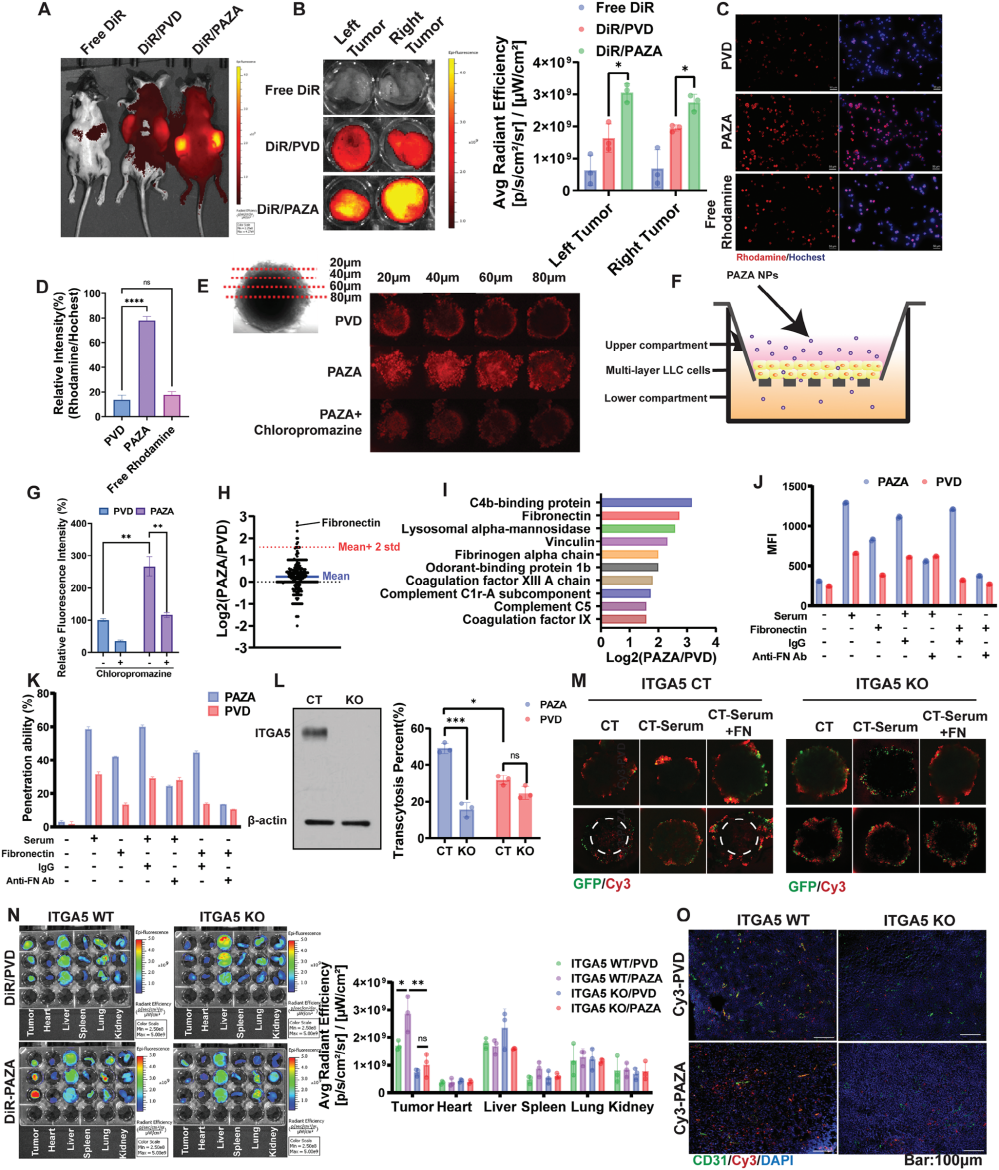
Tumor accumulation and penetration of PAZA carrier through ITGA5 mediated transcytosis. A) In vivo NIR images B) Ex vivo NIR images and quantification of tumors of each mouse treated with DiR‐labeled PVD and PAZA, respectively, at 24 h. (Data are presented as the mean ± S.D, *n* = 3, ^*^ *p* < 0.05, ^**^ *p* < 0.01, ^***^ *p* < 0.001, ^****^ *p* < 0.0001). C) Fluorescence images (Magnification = 40x, bar = 50 µm) and D) quantification of cellular uptake of Rhodamine‐labeled PVD and PAZA by LLC cells at 6 h. (Data are presented as the mean±s.d, *n* = 3, ^*^ *p* < 0.05, ^**^ *p* < 0.01, ^***^ *p* < 0.001, ^****^ *p* < 0.0001). E) Confocal z‐stack images of LLC tumor cell spheroids after 18 h incubation with Rhodamine‐labeled PVD and PAZA NPs, or Rhodamine‐labeled PAZA NPs that had been pretreated with endocytosis inhibitor chlorpromazine. F) Illustration of in vitro transwell assay in multilayer LLC cells and G) Relative fluorescence intensity of lower chamber medium after 6 h incubation with rhodamine‐labeled PVD and PAZA (with or without pretreatment of endocytosis inhibitor chlorpromazine for 1 h) at upper chamber. The fluorescence intensity of each group was compared with the fluorescence intensity of PVD without chlorpromazine treatment group. (Data are presented as the mean ± S.D, *n* = 3, ^*^ *p* < 0.05, ^**^ *p* < 0.01, ^***^ *p* < 0.001, ^****^ *p* < 0.0001). H) Difference of various protein amounts in the protein corona on PVD & PAZA. I) Top ten enriched proteins in protein corona in PAZA compared to PVD. J) Quantification of cellular uptake by flow cytometry of Cy3‐labeled PVD and PAZA by LLC cells with blank medium or medium with various supplements (*n* = 3). K) Transwell assay of transmigration of Cy3 labeled PAZA or PVD across multilayer LLC cells with blank medium or medium with various supplements, respectively (*n* = 3). L) Characterization of ITGA5 knockout cell line and transwell assay of transmigration of Cy3 labeled PAZA or PVD across multilayer control LLC or ITGA5 KO LLC cells. (Data are presented as the mean±s.d, *n* = 3, ^*^ *p* < 0.05, ^**^ *p* < 0.01, ^***^ *p* < 0.001, ^****^ *p* < 0.0001). M) Confocal z‐stack images of middle sections of ITGA5 Control or KO LLC‐GFP spheroids after 18 h incubation with Cy3‐labeled PAZA or PVD with serum (CT) without serum (CT‐Serum) or FN supplement in the medium without serum (CT‐Serum+FN). N) Ex vivo imaging of ITGA5 WT or KO mice and quantification of *ex vivo* imaging at 24 h following i.v. administration of DiR‐loaded, Cy3‐labeled PAZA or PVD. Data are presented as the mean ± S.D, *n* = 3, ^*^ *p* < 0.05, ^**^ *p* < 0.01. O) Fluorescence images of frozen tumor core sections from ITGA5 WT or KO tumor grown on ITGA5 WT or KO mice at 24 h after treatment with Cy3‐labeled PVD and PAZA NPs. CD31 was stained with FITC‐labeled antibody to show the blood vessels. (Magnification = 20x, Bar = 100 µm).

Utilizing an LLC mouse tumor model, we analyzed the tumor‐targeting efficiency of PAZA carrier through NIR imaging and compared it to the larger PVD NPs (**Figure** **2**A). Notably, tumors treated with DiR/PAZA displayed more fluorescence signals compared to DiR/PVD‐treated tumors, which was further supported by the quantification (Figure 2B). The LLC tumors treated with DiR/PAZA and DiR/PVD NPs were further frozen sectioned for penetration study. More DiR signals were observed in the core of tumors treated with DiR/PAZA NPs compared to tumors treated with DiR/PVD NPs, indicating the superior penetration capacity of PAZA carrier in the LLC tumor core (Figure S10, Supporting Information).

To investigate the underpinning mechanism behind the enhanced tumor penetration of PAZA, we initially assessed its uptake by tumor cells and endocytosis pathway with PVD as a control. Our data revealed that PAZA was more effective in internalization into LLC cells compared to the larger PVD NPs (Figure 2C,D). By using different inhibitors of endocytic pathways (Table S2, Supporting Information),^[^ ^18^ ^]^ we found that the cellular uptake of both PAZA and PVD carriers was significantly decreased after preincubation with chlorpromazine, suggesting that both of them were taken up by LLC cells mainly through clathrin‐mediated endocytosis (Figure S11, Supporting Information). The 3D cell spheroids model showed that smaller PAZA was capable of penetrating deeper within tumor spheroids than the larger PVD particles (Figure 2E). Such deep penetration was significantly suppressed by chlorpromazine, indicating that endocytosis may also play a role in tumor penetration. Then, we investigated if the deep tumor penetration of PAZA is mediated by transcytosis pathway via an in vitro transwell assay in a multi‐layer LLC tumor cells model (Figure 2F). By determining the fluorescence intensity in the medium of lower chamber, we found that PAZA carrier was more effective in penetrating through multilayer tumor cells than PVD carrier (Figure 2G). In addition, the penetration of PAZA was greatly inhibited by preincubation with chlorpromazine, indicating the PAZA carrier penetrated deeply into the core of the tumor primarily through the clathrin‐mediated transcytosis rather than paracellular transport due to its smaller size. We also conducted another transwell study in which cells were seeded in both chambers and the labeled NPs were added to the upper chamber. It was apparent that PAZA treatment led to more fluorescence signals in the cells seeded in the lower chamber compared to PVD NPs (Figure S12, Supporting Information), further supporting the notion that the PAZA NPs were effectively endocytosed and then capable of reaching other cells in the deeper layers through transcytosis.

### 1.3 The Role of Fibronectin‐ITGA5 in Mediating Tumor Cell Transcytosis

To elucidate the mechanism underlying the effective transcytosis mediated by the ligand‐free, ultrasmall NPs compared to the large‐sized counterpart, we evaluated how PAZA and PVD engage with the initial biological milieu they encounter upon intravenous administration. Accumulating evidence demonstrated the tendency for most NPs to acquire a coating of serum protein corona post intravenous injection even when using an antifouling polymer such as PEG.^[^ ^19^ ^]^ The PC imparts the NPs with a new biological identity, intricately molding NP interactions within complex biological systems. To study whether PAZA and PVD interact with serum differently and whether such difference contributes to their tumor targeting and penetration efficiency, we conducted proteomics analysis of the protein corona of PVD and PAZA NPs following the exposure to mouse serum. We compared the relative abundance of each component of protein corona on both PAZA and PVD by calculating the PAZA/PVD ratio, as we considered the PAZA/PVD ratio would fit a normal distribution and those outlier proteins (> 2σ) indicate the specific enrichment on PAZA protein corona. Fibronectin (FN) was significantly more enriched in the protein corona of PAZA as opposed to that of PVD (Figure 2H,I). Moreover, it shows considerable absolute abundance (ranked 30th of 393 proteins) compared to other top enriched proteins (e.g., C4b‐binding protein, ranked 102) on the PAZA NPs (Table S3, Supporting Information). The prevalence and substantial enrichment of FN make it a more promising candidate for further investigation. Fibronectin, a crucial glycoprotein, facilitates cell adhesion, migration, and signaling.^[^ ^20^ ^]^ It possesses specific binding sequences such as the RGD motif (arginine‐glycine‐aspartic acid) that can selectively interact with the integrin receptors such as ITGA5 that are significantly upregulated on tumor cells and tumor endothelial cells.^[^ ^21^ ^]^ To investigate if FN in the serum indeed plays a role in the tumor targeting of PAZA, we initially assessed how the serum proteins, especially FN affect the interactions of PAZA or PVD with cultured tumor cells by both fluorescence microscopic examination (Figure S13, Supporting Information) and quantitative flow analysis (Figure 2J; Figure S14, Supporting Information). There was significantly more cell binding/uptake of PAZA NPs compared to PVD NPs in a study with a complete medium. However, the cellular interaction of both NPs was significantly inhibited when a serum‐depleted medium was used. The decreases in cellular uptake were rescued for both NPs, especially PAZA NPs, when FN was added to the serum‐free medium. Importantly, the cellular uptake of PAZA NPs in a complete medium or FN‐supplemented medium was significantly attenuated by ITGA5‐specific antibody but not by a control IgG. These data suggest a role of FN/ITGA5‐specific interaction in mediating the endocytosis of PAZA NPs by tumor cells.

Similar to uptake study, the transcytosis ability was significantly inhibited by depletion of serum or ITGA5‐specific antibody (Figure 2K), suggesting that FN/ITGA5 interaction also contributed significantly to the effective tumor transcytosis. Notably, the NPs exhibited slightly lower penetration ability in the presence of FN compared to that in the serum, indicating that other proteins are also involved in the tumor targeting and penetration of PAZA but to a much lesser extent. Figure 2L shows that KO of ITGA5 led to a drastic decrease in the transcytosis of PAZA NPs. Moreover, in tumorsphere studies with wild‐type LLC cells, PAZA's penetration was hindered by serum deprivation but was partially restored with fibronectin addition, emphasizing its key role in tumor penetration. However, the ITGA5 KO tumor sphere nearly abolished this effect, even when fibronectin was reintroduced (Figure 2M), further confirming a critical interplay between fibronectin and ITGA5 in mediating efficient tumor penetration of PAZA. To establish the in vivo significance of the above studies, the in vivo distribution of PVD and PAZA was also evaluated in mice bearing ITGA5 KO tumors. As shown in Figure 2N,O, the superior tumor accumulation and penetration of PAZA over PVD were abolished when ITGA5 was knocked out in tumors. Taken together, our data suggest that the superior tumor targeting and penetration of PAZA NPs was attributed to the in situ formation of FN‐enriched protein corona after intravenous administration, which promoted transcytosis in the tumor tissue through FN‐ITGA interaction. It is possible other serum proteins might also be involved in tumor targeting and penetration of PAZA NPs, which warrants more studies in the future.

### 1.4 PAZA Carrier Sensitized HR‐Proficient Cancer to BMN

**Figure 3:**
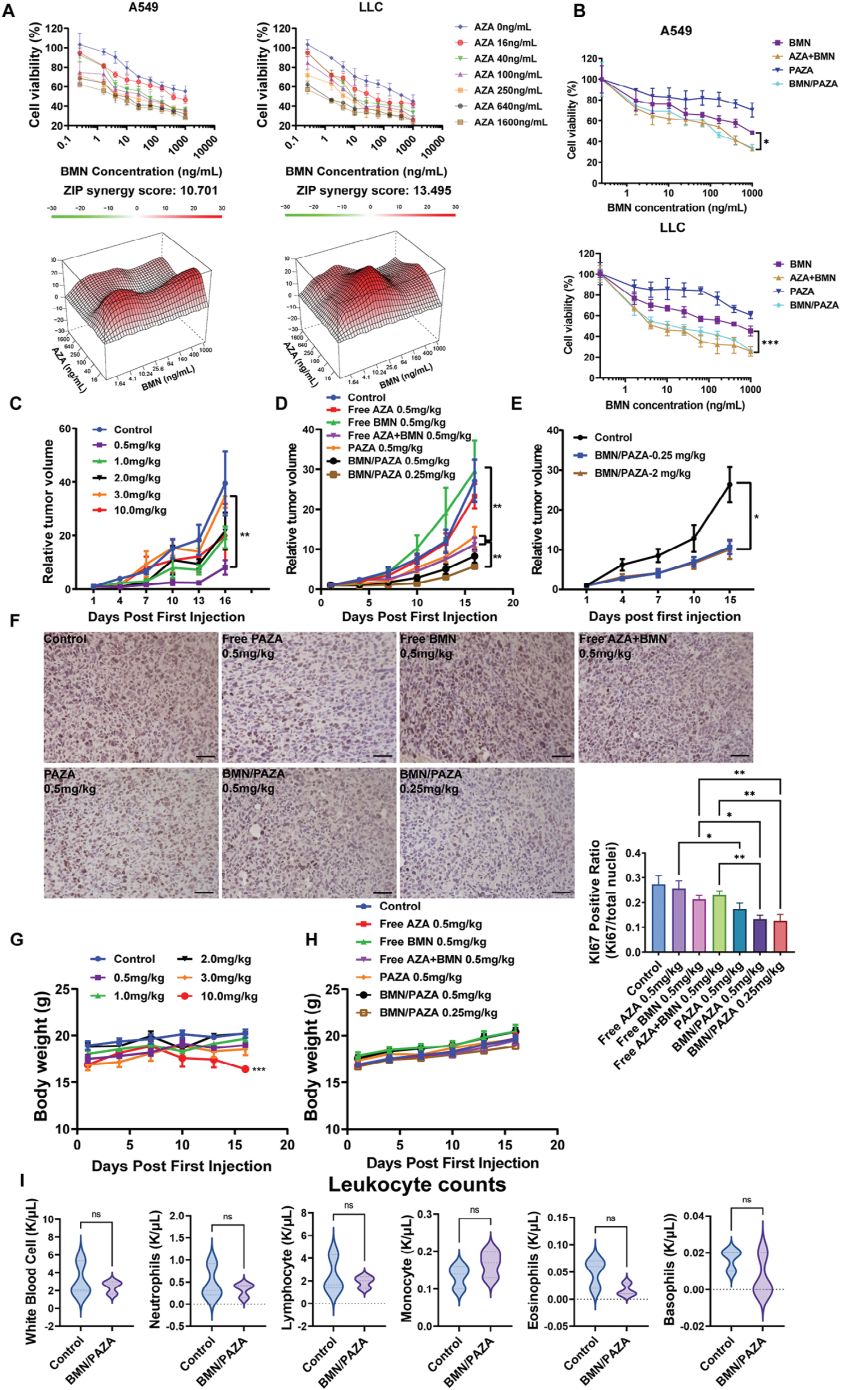
BMN/PAZA combination synergistically inhibited tumor growth. A) Synergy score of various AZA and BMN dosages on A549 and LLC cell lines. Synergy score was calculated based on MTT cytotoxicity assay. (*n* = 5 biologically independent samples, and the data are presented as the mean±S.D.) B) MTT cytotoxicity assay of various formulations in A549 and LLC cell lines (*n* = 6 biologically independent samples, and the data are presented as the mean ± s.d. ^*^ *p* < 0.05, ^**^ *p* < 0.01, ^***^ *p* < 0.001) C–E) In vivo therapeutic effect in the LLC tumor model. C) Relative tumor volume changes of the C57BL/6 mice treated with BMN/PAZA at the different BMN and AZA dosages (the dosage of BMN and AZA is the same, *n* = 3 biologically independent samples, and the data are presented as the mean±sem. ^*^ *p* < 0.05, ^**^ *p* < 0.01, ^***^ *p* < .001). D) Relative tumor volume changes of the C57BL/6 mice treated with various formulations with free BMN and AZA as controls (*n* = 5 biologically independent samples, and the data are presented as the mean±sem, ^*^ *p* < 0.05, ^**^ *p* < 0.01, ^***^ *p* < 0.001). E) Relative tumor volume changes of the LLC tumor‐bearing nude mice treated with two different BMN/PAZA NPs: BMN/PAZA‐0.25 (0.25 mg k^−1^g of BMN and AZA), and BMN/PAZA‐2 (2 mg k^−1^g of BMN and AZA). (*n* = 4 biologically independent samples and the data are presented as the mean±sem, ^*^ *p* < 0.05, ^**^ *p* < 0.01, ^***^ *p* < 0.001). F) Ki67 staining (Magnification = 20x, bar = 100 µm) and the quantification of tumor tissues from (D) (*n* = 3 biologically independent samples, and the data are presented as the mean±sem, ^*^ *p* < 0.05, ^**^ *p* < 0.01, ^***^ *p* < 0.001). G) The change of body weight in (C). H) The change of mice body weight in (D). I) The parameter of leukocytes in the control group and BMN/PAZA group after five treatments (*n* = 3 biologically independent samples).

Following the mechanistic study of tumor targeting and penetration, we evaluated the therapeutic efficacy of PAZA nanocarrier loaded with BMN673. Free AZA and BMN673 showed a synergistic cell‐killing effect with a combination index (CI<1) in two NSCLC cell lines, LLC and A549, at various dose combinations (**Figure** **3**A). PAZA prodrug carrier showed comparable cytotoxicity compared to free AZA in LLC and A549 cells (Figure S15, Supporting Information). Compared to PAZA and BMN alone, incorporation of BMN into PAZA further increased the cytotoxicity (Figure 3B; Table S4, Supporting Information). Compared to the combination of free AZA and BMN, BMN/PAZA showed similar cytotoxicity.

Figure 3C shows the in vivo therapeutic efficacies of BMN/PAZA formulations with various dosages in LLC tumor‐bearing C57BL/6 mice. Surprisingly, simultaneously decreasing the dose of AZA and BMN led to increased therapeutic efficacy. The formulation showed higher therapeutic effect at a lower dosage (0.5 mg k^−1^g of both AZA and BMN). Then, we evaluated the therapeutic effect of BMN/PAZA formulations with free AZA and BMN as controls (Figure 3D). At a dosage of 0.5 mg k^−1^g for both BMN and AZA, BMN alone showed no effect in inhibiting the tumor growth in BRCA‐proficient LLC tumor model, while free AZA only slightly inhibited the tumor growth. PAZA was more effective in inhibiting tumor growth than free AZA. The combination of AZA and BMN showed higher therapeutic effect than the single drugs, and BMN‐loaded PAZA NPs showed the best therapeutic antitumor efficacy. Figure 3D also shows that reducing the dose of both AZA/BMN to 0.25 mg k^−1^g in BMN/PAZA formulation led to further improvement in antitumor activity. The results were further confirmed by the Ki67 immunostaining (Figure 3F) and H&E staining (Figure S16, Supporting Information) of tumor tissues after various treatments. The tumors treated with a lower dosage of BMN/PAZA formulation (0.25 mg k^−1^g) showed widespread inhibition of cell proliferation and enlarged necrosis area. To further gain insight of the dose‐effect, we evaluated the antitumor activity of BMN/PAZA in LLC tumors grown in nude mice (Figure 3E). In this immunodeficient mouse model, there was no difference between the two groups (2 mg k^−1^g vs 0.25 mg k^−1^g) in tumor growth inhibition, suggesting a role of immune response in the higher therapeutic efficacy for BMN/PAZA formulation at lower dosage (0.25 mg k^−1^g) in immunocompetent mice. In addition, the BMN/PAZA formulation was well tolerated with no significant changes in body weight (Figure 3G,H), serum levels of ALT, AST, and creatinine (Figure S17, Supporting Information), and histology of major organs (Figure S18, Supporting Information). However, there was a slight increase in the ALT and AST levels after treatment with free BMN and AZA combination, indicating that loading BMN into PAZA carrier decreased the potential liver toxicity for the combination therapy. A complete blood count (CBC) test further confirmed the excellent safety profile of BMN/PAZA formulation (Figure 3I; Figure S19, Supporting Information).

### 1.5 PAZA Carrier Produced DNA Repair Dysfunction and Enhanced the DNA Damage Induced by BMN

**Figure 4:**
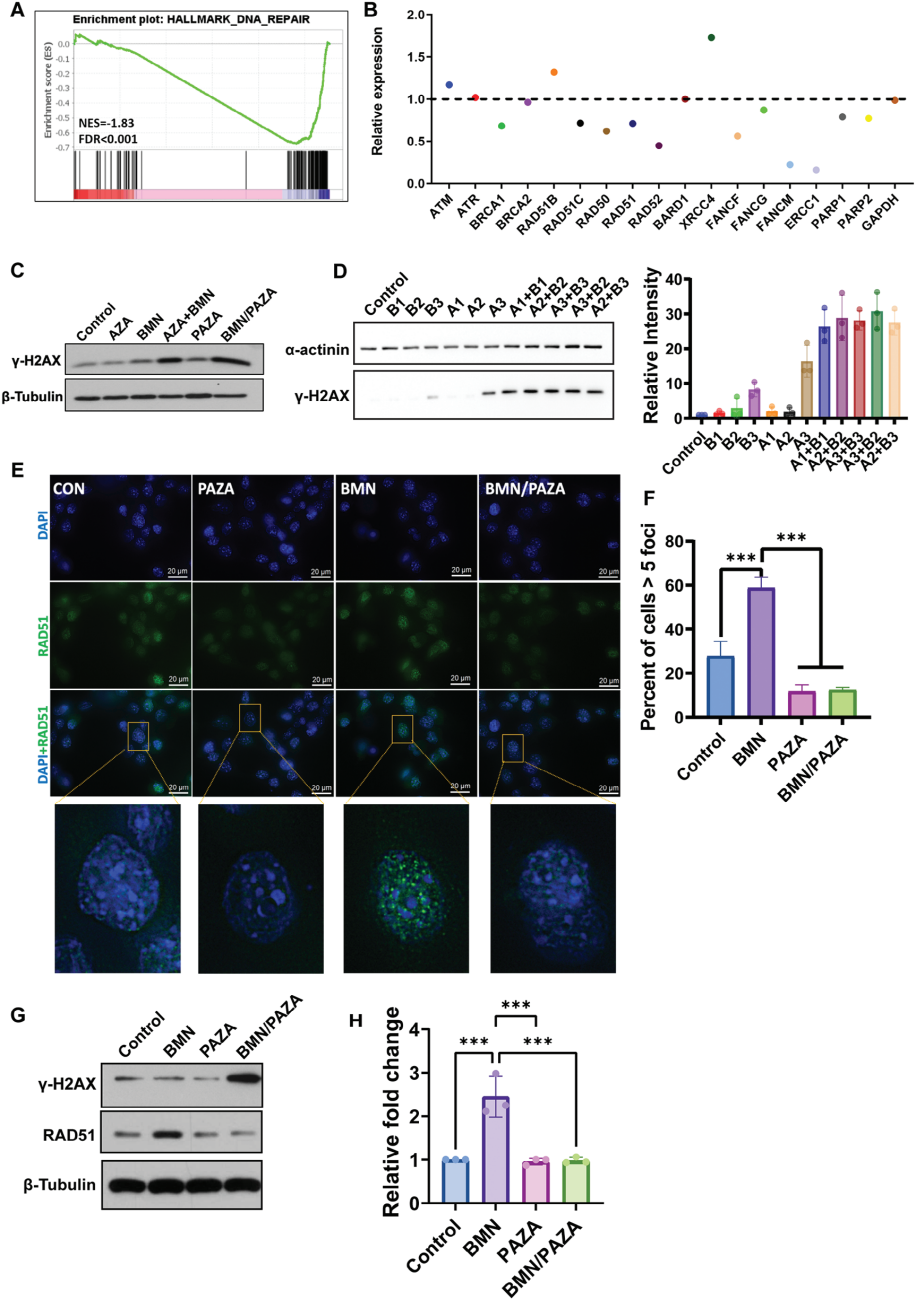
BMN/PAZA combination synergistically induced DNA repair dysfunction and DNA damage. A) Gene Set Enrichment Analysis (GSEA) of transcriptional profiles in DNA repair pathway (BMN/PAZA versus BMN). B) The relative expression level of canonical DNA repair genes in BMN/PAZA treated group (compared to BMN treated group). C) The γ‐H2AX expression levels in LLC cells after various treatments at the BMN dose of 500 nm (BMN: PAZA = 1: 20). D) The γ‐H2AX expression levels and densitometry quantification in LLC cells treated with different doses of free AZA A) and free BMN B). B1: 40 ng mL^−1^ BMN, B2: 200 ng mL^−1^ BMN, B3: 1000 ng mL^−1^; A1, A2, A3 are the AZA concentrations corresponding to B1, B2, B3. (*n* = 3 biologically independent experiment. Data are presented as the mean±S.D.). E) Fluorescence images of RAD51 foci (green) in LLC cell at 48 h after treatment with PAZA, BMN, and BMN/PAZA (Magnification 100X, Scale bar: 20 µm). F) The percent of Rad51 foci‐positive cells after various treatments. (*n* = 3 biologically independent samples. Mean±S.D, ^*^ *p* < 0.05, ^**^ *p* < 0.01, ^***^ *p* < 0.001) G) Protein levels of RAD51 and γ‐H2AX in tumor tissues after various treatments. H) Densitometry quantification of RAD51 signal in (G). (*n* = 3 biologically independent experiment. Data are presented as the mean±s.d, ^*^ *p* < 0.05, ^**^ *p* < 0.01, ^***^ *p* < 0.001).

**Figure 5:**
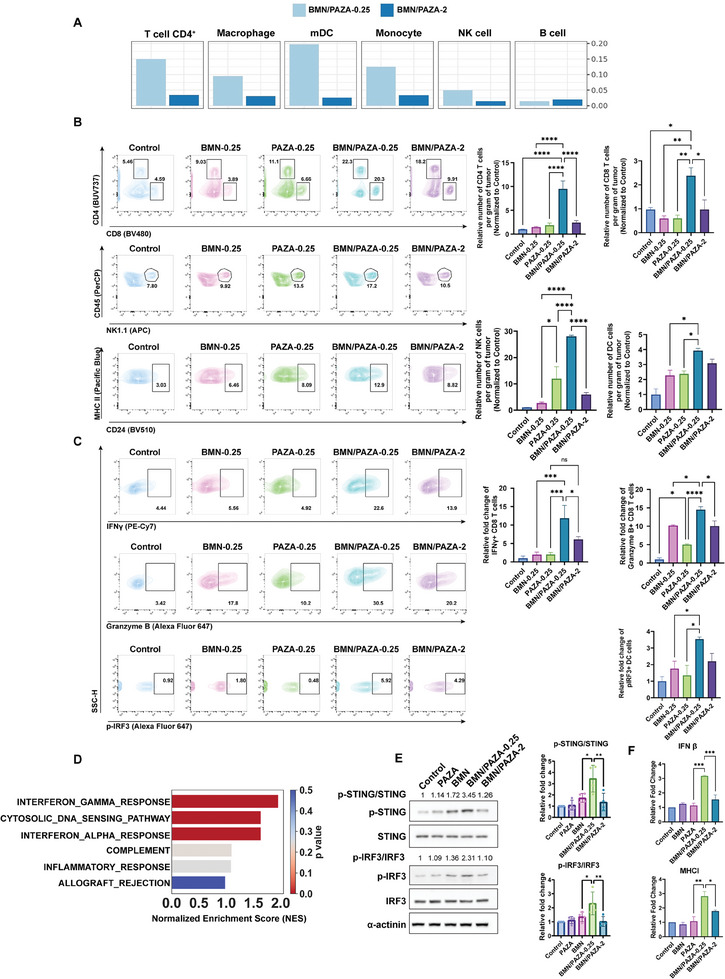
The anti‐tumor immune response elicited by BMN/PAZA combination is dose‐dependent. A) Immune cell infiltration profiles predicted by CIBERSORT based on RNA‐seq data. B) Representative flow cytometric analysis and the quantification for the relative abundance of CD8^+^ T‐cell, CD4^+^ T‐cell, NK cell, and dendritic cell (DC) in tumor tissue after various treatments (*n* = 3, data are presented as the mean±s.e.m, ^*^ *p* < 0.05, ^**^ *p* < 0.01, ^***^ *p* < 0.001). C) Representative flow cytometric analysis and the quantification for the relative abundance of IFNγ^+^CD8^+^ T‐cell, Granzyme B^+^CD8^+^ T‐cell, pIRF3^+^ DC in tumor tissue after various treatments (*n* = 3, data are presented as the mean±s.e.m, ^*^ *p* < 0.05, ^**^ *p* < 0.01, ^***^ *p* < 0.001). D) Gene Set Enrichment Analysis (GSEA) of transcriptional profiles of BMN/PAZA‐0.25 versus BMN in multiple immune‐related pathways. E) The protein levels of p‐STING, STING, p‐IRF3, and IRF3 in mouse LLC tumor tissues after various treatments (*n* = 3 independent experiments, data are presented as the mean±s.e.m, ^*^ *p* < 0.05, ^**^ *p* < 0.01, ^***^ *p* < 0.001). F) The mRNA levels of IFN β and MHCI (*n* = 3 independent samples, data are presented as the mean±S.D., ^*^ *p* < 0.05, ^**^ *p* < 0.01, ^***^ *p* < 0.001).

To gain mechanistic insight into the enhanced therapeutic efficacy of BMN/PAZA and the dose effect, RNA sequencing (RNA‐Seq) of tumor tissues after various treatments were analyzed. Gene set enrichment analysis (GSEA) showed a downregulation of DNA methylation after treatment of BMN/PAZA compared to BMN, which confirmed the epigenetic modulation effect of PAZA (Figure S20, Table S5, Supporting Information). GSEA also showed that DNA repair pathway was significantly inhibited in BMN/PAZA‐treated tumors versus tumors treated with BMN alone (Figure 4A,B; Table S6, Supporting Information), indicating that delivery of BMN using epigenetic‐regulating carrier PAZA may produce DNA repair dysfunction and enhance the DNA damage levels. To test this hypothesis, the DSB levels in cultured LLC cells after various treatments were first evaluated by Western blot using γ‐H2AX as a DNA damage marker. Free AZA treatment didn't increase the protein expression levels of γ‐H2AX at the dose 400 ng mL^−1^ and free BMN treatment led to a small increase in γ‐H2AX levels in LLC cells (Figure 4C). The combination of free AZA and BMN or incorporation of BMN into PAZA further increased the levels of γ‐H2AX. Similar results were found in A549 cells (Figure S21, Supporting Information). We also found that compared to lower dose of AZA (A1 and A2), a higher dose of AZA (A3) was more effective in inducing DNA damage (Figure 4D). This is likely due to that AZA only works as an epigenetic modifier to inhibit DNA methylation when given at low doses. At high doses, it leads to cell death through inhibition of DNA synthesis instead of DNA hypomethylating effect.^[^ ^22^ ^]^ Compared to lower dose of BMN or AZA alone, the combination of AZA and BMN led to significantly increased γ‐H2AX levels. Although the combination of A3+B3 also led to increased γ‐H2AX levels compared to A3 or B3 alone, it showed similar γ‐H2AX levels as the low dose of drug combinations. These studies indicated that compared to the inhibition effect of DNA synthesis, the epigenetic modulation effect of AZA could effectively synergize with BMN in inducing sufficient DNA damage (e.g., DSBs) while maintaining its immune‐promoting activity. At high doses, AZA largely causes nonspecific toxicity, which may also be attributed to immunosuppression as detailed later (**Figure** **5**B,C).

BMN treatment alone increased RAD51 foci, a surrogate HR repair marker, in LLC cells. However, the incorporation of BMN into PAZA led to a significantly decreased level of RAD51 foci (Figure 4E). This was further confirmed by the quantification of the number of RAD51 foci‐positive cells (Figure 4F) and in vivo RAD51 expression in tumor tissues (Figure 4G,H), indicating that BMN treatment induced HR response and DNA repair, which could be reduced by PAZA treatment. All data above indicated that the enhanced synthetic lethality of BMN/PAZA was attributed to the DNA repair dysfunction that was brought by PAZA combination treatment.

### 1.6 BMN/PAZA Increased Innate and Adaptive Antitumor Immune Responses

As indicated by Figure 4C,E, the antitumor immune response may contribute to the better therapeutic efficacy of BMN/PAZA at a lower dose in an immunocompetent murine model. As an initial step to test this hypothesis, we used CIBERSORT ^[^ ^23^ ^]^ to analyze our RNA‐seq data to evaluate the predicted infiltration of immune cells in the tumors treated with formulations at different doses (Figure 5A). Treatment with BMN/PAZA at a lower dose (0.25 mg k^−1^g) led to significant increases in tumor infiltration of multiple immune cell subsets, such as CD4 T‐cell, dendritic cell (DC), and natural killer (NK) cell. In addition, various immune‐related gene sets were enriched in the tumors compared to BMN alone or PAZA. (Figure 5D; Figure S22, Tables S7–11, Supporting Information). It is also apparent from Figure 5A that increasing the dose to 2 mg k^−1^g led to significant decreases in the numbers of these immune cells despite that this dose was well tolerated as shown in other toxicity profiles (Figure 3G).

Flow cytometry showed that BMN or PAZA treatment led to an increase in the numbers of NK and DC cells, but no significant changes in CD4 and CD8 T cells in the tumors (Figure 5B). In comparison, BMN/PAZA formulation at the same dose (0.25 mg k^−1^g for BMN and AZA) led to significant increases in the numbers of these immune cells in the tumor tissues. Furthermore, compared to BMN or PAZA, BMN/PAZA combination treatment led to significant increases in the population of IFNγ^+^ CD8 T cells and Granzyme B^+^ CD8 T cells, suggesting significant activation of functional T‐cells (Figure 5C), which was also seen in IFNγ gene set enrichment (Figure 5D; Table S10, Supporting Information). It should be noted that the improvement in the immune profile of BMN/PAZA was significantly diminished at a higher dose (Figure 5B,C). These data, together with the data on decreased therapeutic efficacy of BMN/PAZA at the higher dose (Figure 3C,D), strongly support an important role of immune response in the overall therapeutic efficacy of BMN/PAZA.

The superior antitumor activity may be attributed to the enhanced delivery of both BMN and PAZA and/or a synergy between PAZA and BMN. To elucidate the role of the latter mechanism, a “pharmacologically inert” carrier of comparable delivery efficiency (PCyt) was prepared by replacing AZA with cytidine (Figure S23, Supporting Information). Compared to BMN treatment, there were no significant changes in the relative number of CD4, CD8, and NK cells in the tumors after BMN/PCyt treatment. In comparison, BMN/PAZA led to a significant increase in these immune cells in the tumors (Figure S24, Supporting Information), suggesting an important role of the synergy of PAZA and BMN in the overall antitumor activity of BMN/PAZA.

RNA‐seq analysis of BMN/PAZA‐treated tumors revealed significant gene enrichment in the cytosolic DNA sensing pathway and Type I IFN pathway compared to BMN group (Figure 5D; Tables S11 and S12, Supporting Information). Western blot further confirmed that loading BMN into PAZA carrier activated the STING pathway by increasing the protein expression of the phosphorylated STING (pSTING) and the downstream phosphorylated IRF‐3 (pIRF‐3) in the tumors (Figure 5E). BMN/PAZA formulation was also more effective in eliciting the STING activation at a lower dosage. We also assessed the IFNβ and MHCI expression levels after various treatments (Figure 5F; Figure S25, Supporting Information). While BMN or PAZA alone was not effective in inducing the expression of IFNβ and MHCI, BMN/PAZA showed strong induction of both IFNβ and MHCI, which was also more drastic at a lower dosage. These data indicated that BMN/PAZA combination showed significant synergy in eliciting innate immune response that subsequently further boosts the adaptive immune responses. In addition to providing an effective strategy for improving the efficacy of synthetic lethality therapy for HR‐proficient NSCLC, BMN/PAZA shall represent a safe therapy due to its unique dose‐response profile. The safety shall also benefit from the improvement in tumor‐selective delivery.

### 1.7 BMN/PAZA Synergized with aPD‐1 in an Orthotopic Model of Lung Cancer

**Figure 6:**
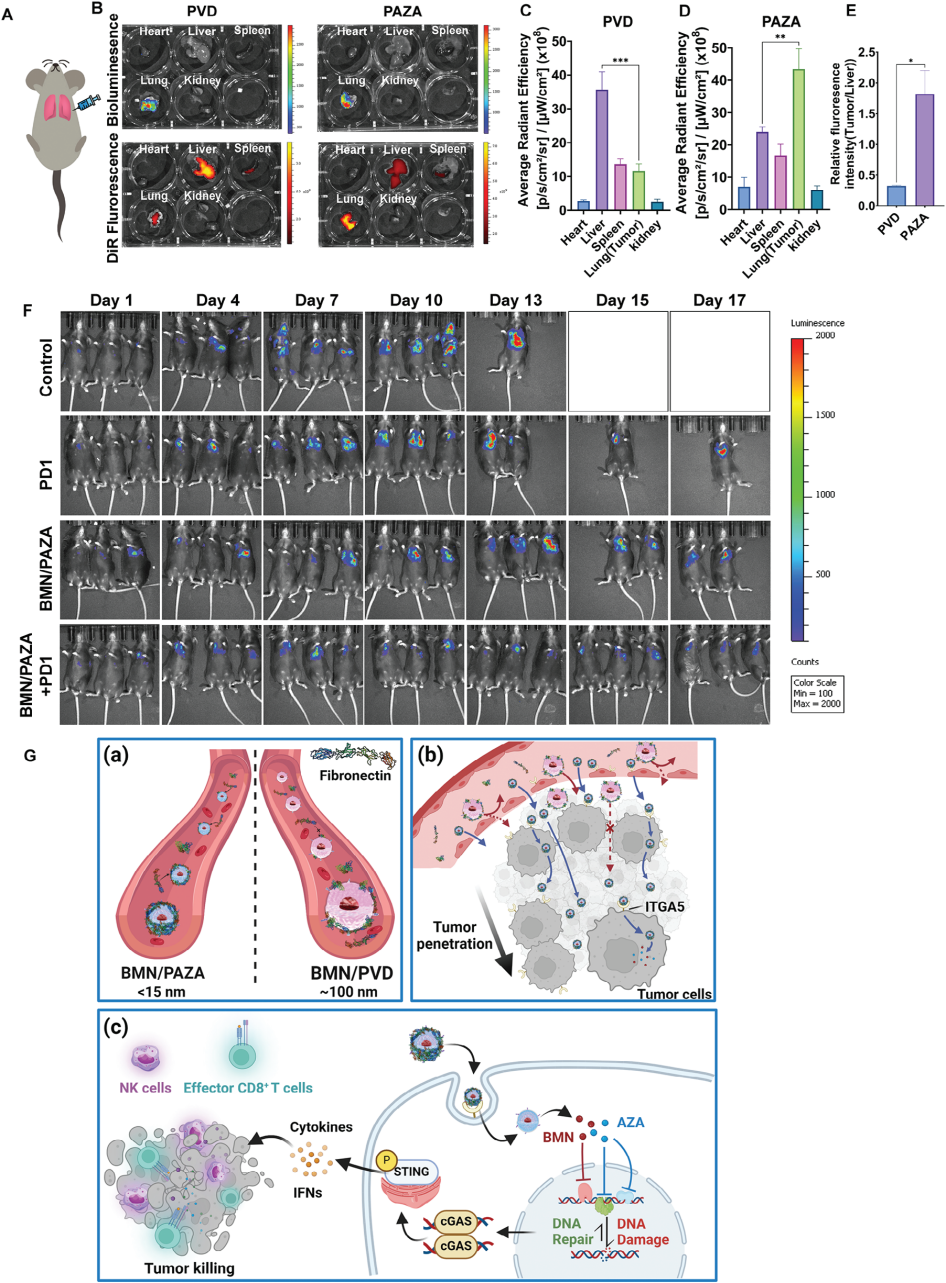
BMN/PAZA in combination with PD‐L1 blockade inhibits tumor growth in the orthotopic model. A) Schematic diagram of establishing the lung orthotopic model. B) Bioluminescence and ex vivo NIR images of major organs and lung orthotopic tumors of each mouse treated with DiR‐labeled PVD and PAZA respectively at 24 h. C–E) Quantification ex‐vivo image of C) PVD, D) PAZA, and E) the relative fluorescence intensity of tumor to the liver in PVD and PAZA treatment group respectively (*n* = 3 independent animals, data are presented as the mean±s.e.m, ^*^ *p* < 0.05, ^**^ *p* < 0.01, ^***^ *p* < 0.001). F) Tumor growth represented by luminescence intensity after PD‐1, BMN/PAZA, or BMN/PAZA+PD‐1 combination treatment. G) Schematic illustration of the formation of fibronectin‐enriched PC on small‐sized BMN/PAZA NPs a), the tumor penetration through ITGA5‐dependent transcytosis of small‐sized BMN/PAZA NPs b), and the tumor inhibition via increased DNA damage and activation of immune response of BMN/PAZA combination c). Systemic administration of BMN/PAZA NP could recruit fibronectin on its surface as protein corona, followed by efficiently targeting and penetrating the tumor deeply through ultra‐small size effect and ITGA5‐dependent transcytosis mechanism. Release of BMN‐673 and 5‐azacitidine inhibited the PARP and the DNMT respectively. The synergistic inhibition induced DNA repair dysfunction and DNA damage. Low‐dose combination treatment could further elicit the cGAS/STING‐based innate immune pathway. The epigenetic modulation of tumor cells and activated innate immune together contributed to the activation of T‐cell and NK cells. The figure is created with BioRender.com.

In addition to an overall improvement in the immune landscape, BMN/PAZA treatment led to upregulation of PDL1 expression (Figure S26, Supporting Information) in tumor cells, indicating the potential benefit from combination therapy with PD‐1 antibody (aPD‐1).^[^ ^24^ ^]^ To examine the therapeutic efficacy of BMN/PAZA+aPD‐1 combination, we established an orthotopic lung cancer model by transplanting LLC‐Luc into the lung of C57BL/6 mice (**Figure** **6**A). Compared to larger PVD nano‐carriers, PAZA showed robust tumor targeting in lung orthotopic tumors (Figure 6B–E). The control mice and PD1 treated group showed rapid cancer progression, which was reflected by in vivo imaging of LLC‐Luc signal (Figure 6F). In comparison, the BMN/PAZA treated group showed a stronger tumor growth inhibition effect. The BMN/PAZA + aPD‐1 combination‐treated group showed repressed tumor growth and recession of tumor area after 2 weeks of treatment, demonstrating the enhanced synergy in the overall antitumor activity in the orthotopic lung tumor model.

## 2 Discussion

Suboptimal tumor targeting and penetration remain significant challenges in the clinical translation of nanomedicine. To address these challenges, two primary strategies have emerged. The first strategy is to design small‐sized nanocarriers that can achieve better tumor targeting and penetration presumably through benefiting more from the leaky blood vessels and cellular gaps.^[^ ^25^ ^]^ In addition, nanoparticles with a particle size below 20 nm have been reported to be more advantageous in overcoming interstitial fluid pressure (IFP) and extracellular matrix (ECM), which is beneficial to achieve deep tissue penetration.^[^ ^26^ ^]^ Another strategy is to incorporate a targeting ligand to improve specific tumor cell binding and enhance transcytosis through endothelial cells and tumor cells.^[^ ^27^ ^]^ However, while these strategies have been explored, the design of NPs often overlooks the influence of the protein corona on in vivo behaviors. PC was reported to mask intended targeting ligands of the nanocarriers, resulting in nonspecific cellular interactions.^[^ ^28^ ^]^ However, recent studies have shown that certain proteins within the corona, such as albumin, transferrin, and apolipoprotein, can potentiate the targeting and therapeutic capabilities of NPs.^[^ ^29^ ^]^ This underscores the need to consider PC during the nanocarrier design and develop strategies that promote the adsorption of desirable proteins while minimizing the nonspecific adsorption of undesirable ones.

In our study, the conjugation of AZA to the PVD backbone altered the PC composition on the NPs. Specifically, FN became more enriched in the corona of PAZA NPs. This is unlikely due to the small size effect of PAZA NPs, as FN was also more enriched in the corona of PAZA NPs compared to another previously reported ultra‐small nanoparticle, PGEM ^[^ ^25^, ^30^ ^]^ (Figure S27, Supporting Information). PGEM is a polymer that has AZA replaced with gemcitabine and displays a size comparable to that of PAZA. Thus, FN enrichment may be a result of changes in the surface chemistry of the NPs rather than their particle size. The precise mechanisms that lead to FN's affinity for PAZA's surface merit further investigation.

The FN interacts with the ITGA5 receptor on tumor cells, facilitating increased tumor cellular uptake and transcytosis of PAZA NPs, leading to enhanced tumor penetration (Figure 6G). This work represents the first to show that small, ligand‐free NPs can gain tumor‐targeting properties following i.v. administration via recruiting tumor‐homing protein FN. In addition to providing a simple and effective tumor‐targeting system, this study may have a broad implication in understanding the interaction of NPs with biological systems and the potential biological consequences. We acknowledge that ITGA5 on tumor endothelial cells shall likely contribute to the tumor targeting of PAZA NPs, which will be further studied in the near future.

In addition to excellent tumor targeting and penetration, PAZA nanocarrier shows excellent loading capacity for both hydrophilic AZA and hydrophobic PARPi BMN. Molecular dynamics and experimental validation reveal the mechanism behind the high drug loading capacity of the small nanocarrier, with the pyrimidine ring of AZA contributing significantly to BMN loading through π‐π interactions. Moreover, the pharmacological combination of PAZA and BMN showed a strong synergistic effect through the inhibition of DNA repair and the activation of both innate and adaptive immune responses. More importantly, BMN/PAZA formulation was more effective in tumor inhibition at lower doses due to the stronger immune activation, which denotes a wider therapeutic window with low toxicity (Figure 6G). Furthermore, BMN/PAZA increased the immunogenicity and potentiated the response to aPD‐1 treatment, expanding the clinical application of aPD‐1 in those tumors with poor immune checkpoint blockade response. All results together suggested an exciting possibility for expanding PARPi in HR‐proficient NSCLC by our small PAZA nanocarrier.

## 3 Experimental Section

### Reagent

4‐Vinylbenzyl chloride, 4,4′‐dithiodibutyric acid, triethylamine, 4‐Cyano‐4‐(phenyl‐carbonothioylthio)pentanoic acid, poly(ethylene glycol)methyl ether methacrylate (average Mn = 950), 2,2‐Azobis (isobutyronitrile) (AIBN), Dulbecco's Modified Eagle's Medium (DMEM), RPMI 1640 Medium, trypsin‐EDTA solution, 3‐(4,5dimethylthiazol‐2‐yl)−2,5‐diphenyl tetrazolium bromide (MTT), D‐Luciferin were purchased from Sigma‐Aldrich (MO, U.S.A), BMN673 and 5‐Azacytidine were purchased from MedChemExpress LLC (NJ, USA). Antibodies for western blot, IHC, immunostaining, and flow cytometry were listed in Table S13 (Supporting Information).

### Cell Lines and Animals

LLC murine NSCLC cancer cell line and A549 human NSCLC cell line were obtained from ATCC (Manassas, VA). They were cultured in DMEM medium supplemented with 10% FBS and 1% penicillin/streptomycin at 37 °C in a humidified atmosphere with 5% CO2.

ITGA5 KO cell line was generated by using CRISPR technology. LLC cells were infected with the lentivirus packaged by ITGA5‐All‐in‐one lentiviral sgRNA‐CRISPR‐Cas9 plasmid encoding EGFP and puromycin resistance (Horizon Discovery Ltd., Cambridge, UK). The successfully knocked‐out cells were selected by cell sorting of EGFP+ and ITGA5 KO populations. Cells were further confirmed by western blot analysis for the lack of ITGA5 expression.

Female C57BL/6 mice (4–6 weeks) (Strain #:000664) were purchased from Jackson Laboratory (ME, U.S.A). All animals were housed under pathogen‐free conditions according to AAALAC (Association for Assessment and Accreditation of Laboratory Animal Care) guidelines. All animal‐related experiments were performed in full compliance with institutional guidelines and approved by the Animal Use and Care Administrative Advisory Committee at the University of Pittsburgh. under Protocol #: 21099779. Mice were housed at an ambient temperature of 22 °C (22–24 °C) and humidity of 45%, with a 14/10 day/night cycle (on at 6:00, off at 20:00), and allowed access to food ad libitum.

### Polymer Synthesis, Chemical Characterization, and Molecular Simulation

Detailed information on the polymer synthesis, characterization, and modeling is available in Supplementary methodological information.

### Preparation and Characterization of Drug‐Loaded Micelles

Blank micelles and BMN‐loaded micelles were prepared via a film hydration method. Briefly, BMN solution (10 mg mL^−1^ in methylene chloride) was mixed with PVD or PAZA polymer (20 mg mL^−1^ in methanol) at different carrier/drug weight ratios. The solvent was removed by nitrogen flow to produce a thin film of carrier/drug mixture, which was further dried in a vacuum for 2 h to remove any remaining solvent. Then the thin film was hydrated and gently vortexed in PBS to form the final formulation. Particle sizes, polydispersity, and morphology were measured by DLS (Nano‐ZS 90, Malvern Instruments, Malvern) and transmission electron microscopy (TEM). CMC was measured using Nile red as a fluorescence probe.^[^ ^31^ ^]^ Drug loading capacity and efficiency of BMN were measured by a Waters e2695 HPLC system equipped with a Waters 2489 UV detector according to a published chromatography condition.^[^ ^32^ ^]^

### Stability and Drug Release Kinetics

The stability of the BMN/PAZA drug‐loaded micelles was measured both in PBS solution and after lyophilization. For lyophilization stability study, 1 mL of BMN/PAZA micelle solution was lyophilized after size measurement. The lyophilized powder was re‐dissolved by 1 mL nano water to regenerate the BMN/PAZA drug‐loaded micelle solution for size measurement. For long‐term stability study, the micelle solution was re‐measured for size after standing 4 weeks under room temperature.

BMN in vitro drug release study was conducted by dialysis method. Briefly, 1 mL of BMN/PAZA micelles containing 6 mg of BMN in PBS buffer were placed in a clamped dialysis bag and immersed in 25 mL of 0.1 m PBS buffer solution containing 0.5% (w/v) Tween 80 with 10 mm GSH, 10 µM GSH or without GSH. Each group was triplicated. The experiment was performed in an incubation shaker at 37 °C at 100 rpm. At selected time intervals, both 10 µL BMN/PAZA micelles solution in the dialysis bag and 1 mL medium outside the dialysis bag were withdrawn while the same amount of fresh medium was added for replenishment. The BMN concentration was determined by HPLC as described above.

AZA release study was also conducted similarly. PAZA micelle (20 mg) in PBS buffer were placed in a clamped dialysis bag and immersed in 50 mL of 0.1 m PBS buffer solution with 10 mm GSH, or without GSH. For stability assay, 20 mg of blank PAZA micelle in 100 uL plasma or tumor lysate (100 mg mL^−1^) were placed in a clamped dialysis bag and immersed in 50 mL of 0.1 m PBS buffer solution. Each group was triplicated. The experiment was performed in an incubation shaker at 37 °C at 100 rpm. At selected time intervals, 10 mL of medium outside the dialysis bag was withdrawn while the same amount of fresh medium was added for replenishment. The withdrawn medium was lyophilized and the AZA was extracted by 90% methanol. The elution condition is 0.1% TFA: Methanol: Acetonitrile = 95:2.5:2.5.

Intracellular release of AZA was detected by LC‐MS. The LLC cells were incubated with 10 mg of PAZA for 6, 12, 24, 48, and 72 h respectively. Cells were lysed and extracted by 50% methanol. 100 µL of the sample was transferred to a tube, to which 100 µL methanol containing the internal standard (gemcitabine) was added. The samples were then vortexed and centrifuged for 10 min at 10 000 x g. The AZA concentration was detected by LC‐MS/MS. Chromatographic separation was performed using a Thermo Hypersil GOLD C18 column (2.1 × 100 mm, 1.9 µm). An isocratic (50:50) gradient was used for AZA with a total runtime of 3 min using the mobile phases water with 0.1% formic acid (A) and acetonitrile (B).

### In Vitro Cytotoxicity Assay

Cytotoxicity assay was performed on BRCA‐proficient NSCLC cell lines LLC and A549. The cytotoxicity of AZA, BMN673, AZA plus BMN, PAZA carriers alone, and PAZA/BMN with different doses were examined by MTT assay. Cells were seeded in 96‐well plates at a density of 5000 cells well for 24 h. Then, the medium was replaced with fresh medium containing different formulations every 24 h for 96 h. After incubation, the medium in each well was replaced with 0.2 mL of fresh medium containing 0.5 mg mL^−1^ MTT, and incubated for another 3 h. The medium in each well was further replaced with 0.1 mL DMSO to dissolve the formazan crystals. The absorbance in each well was measured at 562 and 620 nm using a microplate spectrophotometer. Cell viabilities were calculated from the following equation, where OD is the optical density of the sample or control at the indicated wavelength:

(1) $$\mathrm{Cell}\;\mathrm{viability}\;\left(\%\right)=\frac{\mathrm{ODsample},\;562\mathrm{nm}-\mathrm{ODsample},620\mathrm{nm}}{\mathrm{ODcontrol},562\mathrm{nm}-\mathrm{ODcontrol},620\mathrm{nm}}$$

Synergy was calculated through SynergyFinder.^[^ ^33^ ^]^

### Cellular Uptake and Endocytosis Pathway Analysis

For the cellular uptake study, the LLC cells were incubated with rhodamine‐labeled PAZA and PVD NPs, respectively, for 6 h. Then, the culture medium was discarded, and cells were washed with cold saline for three times. The cell nucleus was stained with Hoechst 33342 Cellular, followed by washing with saline for three times. Cellular uptake was measured by fluorescence microscope (BZ‐X800, Keyence). For endocytosis pathway analysis, the cells were preincubated with different endocytosis inhibitors including filipin, chlorpromazine, amiloride, dynasore, cytochalasin D and mβCD, separately for 2 h at the working concentrations that were not toxic to the cells.^[^ ^18^ ^]^ Then, the medium was replaced with fresh medium and treated with rhodamine‐labeled PAZA and PVD NPs (500 ng mL^−1^ rhodamine), respectively. At 6 h after the addition of NPs, the cells were washed with cold saline for three times and analyzed by flow cytometry.

### In Vitro Transwell Assay

For all transwell‐based studies, an in vitro multilayer LLC cell or LLC‐ITGA5 KO cell model was established by seeding the cells in a 0.4 µm diameter microporous membrane (Corning, product no. 3470). Membranes were inserted in 12 well plates followed by incubation for 4 days.^[^ ^34^ ^]^ The lower chamber was added with blank DMEM medium, and the upper chamber was added with DMEM medium containing rhodamine‐labeled PVD or PAZA NPs (500 ng mL^−1^ rhodamine), with or without pretreatment with transcytosis inhibitor chlorpromazine (6 µg mL^−1^) for 2 h. At 10 h after the addition of NPs, the fluorescence intensity of the medium in the basolateral side was determined. In fibronectin‐mediated transcytosis study, similarly, the lower chamber was added with blank DMEM medium, and the upper chamber was first treated with Anti‐ITGA5 antibody or IgG isotype control for 2 h, then added with blank DMEM medium containing rhodamine‐labeled PVD or PAZA NPs (500 ng mL^−1^ rhodamine), with supplement of fibronectin (10 µg mL^−1^) or mouse serum. In a separate study, cells were first seeded in 0.4 µm diameter microporous membrane, followed by incubation for 4 days to develop the multilayer cells. One day before treatment, the lower chamber was replaced with a new plate and seeded with LLC cells. Then, the upper chamber was added with DMEM medium containing rhodamine‐labeled PVD or PAZA NPs (500 ng mL^−1^ rhodamine), respectively. At 6 h after the addition of NPs, the cells were washed with cold saline for three times and analyzed by flow cytometry.

### Cell Spheroid Penetration

WT or ITGA5 KO LLC cells were seeded in a Nunclon Sphera 96 well U‐bottom plate (Thermofisher) at a density of 10 000 cells per well with 6 µg mL^−1^ of collagen I to form a single spheroid per well.^[^ ^35^ ^]^ After 96 h incubation, dense spheroids were formed, which were confirmed by microscope. The cell spheroids were incubated with rhodamine‐labeled PVD or PAZA NPs (500 ng mL^−1^ rhodamine) respectively, with or without pretreatment with transcytosis inhibitor 6 µg mL^−1^ chlorpromazine (6 µg mL^−1^) for 2 h. In a separate study, the cell spheroids were incubated with rhodamine‐labeled PVD or PAZA NPs (500 ng/mL rhodamine) in 10% FBS medium, blank medium, or medium with fibronectin (10 µg mL^−1^) respectively. After 18 h incubation with NPs, the cells were gently rinsed by saline for three times. The penetration ability was observed by a confocal laser scanning microscope with Z stack scanning (CLSM, FluoView 3000, Olympus) at 20 µm intervals from the bottom to the middle of the spheroids.

### Tumor Models

Subcutaneous (s.c.) LLC tumor model was established by injecting LLC cells into the flank of C57BL/6 mice. The Orthotopic LLC tumor model was established by resuspending 1 × 10^6^ luciferase‐expressing LLC cell line (LLC‐Luc) in a 1: 1 mixture of PBS and Growth Factor‐Reduced (GFR)‐Matrigel, followed by orthotopically injecting into the left lateral thorax of C57B6 mice.^[^ ^36^ ^]^ The tumor size and location were monitored and quantified with a noninvasive bioluminescence system (Perkin Elmer IVIS 200 system).

### Tumor Targeting and Tumor Penetration

The tumor‐targeting effect of PAZA was evaluated in both WT/ITGA5 KO LLC model (s.c). and the orthotopic model using a larger PVD carrier as a control. Hydrophobic fluorescence dye DiR was loaded into the PAZA carrier or PVD control polymer at a wt/wt ratio of 40:1 and intravenously injected into the mice for real‐time imaging at the DiR dosage of 1 mg k^−1^g. After 24 h, the mice were imaged by an IVIS 200 system with excitation at 730 nm and emission at 835 nm. The tumor and various organs were then excised for ex vivo imaging following our previous protocol.^[^ ^31^ ^]^ The tumor was then frozen sectioned, and stained with DAPI to label the cell nucleus and the antibody for CD31 to label the vascular endothelial cell. The fluorescence signals in the core of the tumor were examined under Keyence BZ‐X800 fluorescence microscope.

### Therapeutic Study

The LLC tumor‐bearing mice (five mice each group) with tumors of volume ≈50 mm^3^ were randomly categorized into study groups and intravenously (IV) injected with saline, BMN, AZA, PAZA carrier, BMN/PAZA, and free BMN+AZA combination once every 3 days for five times. The tumor volume and body weight were measured every 3 days. After the completion of the experiment, the tumor tissue and major organs were harvested, fixed with 4% (v/v) paraformaldehyde in PBS (PH 7.4), embedded in paraffin, and sectioned into 4 µm slices. Each section was processed for H&E staining and observed under a microscope. Immunohistochemical analysis (IHC) of Ki67 protein, an important biomarker of cell proliferation, was carried out using the labeled streptavidin‐biotin method. Ki67 expression was quantified by calculating the number of Ki‐67 positive cells/total number of cells in five randomly selected areas using ImmuneRatio software.

LLC‐Luc orthotopic model was used to evaluate the combination effect of PAZA/BMN with PD‐1 antibody, and mice were randomly categorized into study groups 10 days after surgery inoculation. PAZA carrier, BMN/PAZA combination (0.25 mg k^−1^g) was intravenously injected once every 3 days. Antimouse PD‐1 antibody (InvivoGen) (100 µg each mouse) was intraperitoneally injected once every 3 days. The tumor volume of the mice was monitored by IVIS‐200 imaging. The 150 mg k^−1^g D‐luciferin was injected intraperitoneally for the bioluminescence imaging of each mouse.

### Toxicity Study—Biochemical Parameters

Blood samples were collected after five injections of saline and BMN/PAZA formulations into tumor‐bearing mice. The serum alanine aminotransferase (ALT), aspartate aminotransferase (AST), and creatinine levels were evaluated as indicators of hepatic and renal function.

### Toxicity Study—Complete Blood Count (CBC)

Blood samples were collected after five injections of saline and BMN/PAZA formulations into tumor‐bearing mice. A blood sample was measured by a CBC analyzer in UPMC Hillman Cancer Center.

### Toxicity Study—Histology Changes

The tissue section of major organs (heart, liver, spleen, lung, and kidney) that was collected after the therapeutic study was processed for H&E staining to test if there is a morphology change to major organs after treatments.^[^ ^37^ ^]^

### Western Blot

The expression levels of γ‐H2AX, RAD51, STING, phosphorated STING, IRF3, and phosphorated IRF3 in cells and tumor tissues after various treatments were evaluated by western blot. The cells and homogenized tumor tissues after various treatments were lysed in RIPA buffer with protease inhibitor and, PhosSTOP phosphatase, followed by quantitated for protein using the BCA Protein Assay Kit. Equal amounts of protein were resolved by SDS‐PAGE. Membranes were blocked in 5% BSA/PBST and incubated with antibodies. Immunodetection was performed using SuperSignal West Pico and Femto Chemiluminescent Substrate. Blot stripping was performed using Restore Western Blot Stripping Buffer according to the manufacturer's guidelines. Protein levels were quantified by densitometric analysis using ImageJ/Fiji. Phosphorylated protein levels were normalized to the total protein band, then to loading control, and expressed as fold change versus control DMSO.

### Immuno‐Fluorescence Staining

DNA repair capability after various treatments was evaluated by determining the RAD51 foci via immunofluorescence staining. Cells after various treatments were washed, fixed, and permeabilized in 0.25% Triton/PBS. Blocking was performed in 5% BSA/0.1% Triton/PBS, followed by incubation with primary antibodies in dilution buffer (1%BSA/0.1%Triton/PBS) (overnight, 4 °C). Cells were then washed in PBS and incubated with secondary antibodies, followed by washing and mounting on coverslips using ProLong Gold antifade reagent with DAPI. Cells were imaged using Keyence BZ‐X800 fluorescence microscope and analyzed using ImageJ/Fiji software. FindFoci ImageJ plugin was used for RAD51 foci quantification, at least five fields were counted for foci number respectively.^[^ ^38^ ^]^

### Analysis of Tumor‐Infiltrating Immune Cells

The immune cell populations in the tumors after various treatments were measured by flow cytometry following the previous protocol.^[^ ^39^ ^]^ Briefly, one day after the last treatment, cell suspensions from the spleens or tumors were filtered, and red blood cells lysed. Single‐cell suspensions were incubated with respective flow antibodies. Zombie dye was used to discriminate between viable and dead cells. Infiltration of various immune cells (CD4^+^, CD8^+^, Treg, natural killer (NK) cells, myeloid‐derived suppressor cells (MDSC), dendritic cells, and macrophages) in tumor tissues, the production of lymphocyte effector molecules (such as IFN‐γ, perforin, and granzyme B) on immune cells, and MHC‐I on tumor cells were determined by multicolor flow cytometric analysis. The expression of phosphorated IRF3 and TBK1 in tumor cells and DC cells was also evaluated by flow cytometry.

### RNAseq Analysis

One day after the last treatment, the tumors were collected for RNAseq, which was performed at the Health Sciences Sequencing Core at Children's Hospital of Pittsburgh. Raw sequence data was analyzed as described in the previously published protocol ^[^ ^40^ ^]^ to generate transcript‐level gene expression. Then gene set enrichment analysis (GSEA) ^[^ ^41^ ^]^ was performed to identify the treatment‐associated alteration in functional pathways. The bulk messenger RNA‐seq data mapped to the mouse genome (GRCm38: https://www.ncbi.nlm.nih.gov/assembly/GCF_000001635.20/) are available in the NCBI to Gene Expression Omnibus with accession number GSE245266.

### Proteomic Analysis

Cy3‐labeled PAZA and PVD were incubated with mouse serum for 6 h. The Sephadex G‐100 column was used to remove unbounded protein. The PAZA or PVD with protein corona was collected and quantified based on the fluorescence intensity. Polymer‐protein corona complexes were then loaded in SDS‐PAGE gel. Gel slice samples were sent to BGI America Mass Spec Service Center for proteomic analysis. Briefly, Gel slices were digested with Trypsin/LysC according to the standard in‐gel digestion protocol.^[^ ^42^ ^]^ Samples were then extracted, dried, and reconstituted for LC‐MS/MS. MS Data was searched against the most updated UniProt Mouse database. Sequest analysis workflow was used to reveal basic protein identification information.

### Statistical Analysis

Data are presented as the mean ± S.D or mean ± s.e,m as indicated in figure legend. Statistical analysis was performed with a two‐tailed Student's *t*‐test for comparison between two groups and one‐way analysis of variance (ANOVA) with Tukey's post hoc test for comparison between multiple groups as indicated in figure legend. Results were considered statistically significant if *p* < 0.05. Prism 10.1.0 (GraphPad Software) was used for data analysis and graph plotting.

## Conflict of Interest

The authors declare the following financial interests/personal relationships which may be considered as potential competing interests: The authors declare the following competing financial interest(s): Song Li serves on the Chief Scientific Officer and holds equity in DUO Oncology. Jingjing Sun is an inventing consultant and equity holder in DUO Oncology.

## Supporting information

Supporting Information

Supplemental Table 1

## Data Availability

The bulk messenger RNA‐seq data mapped to the mouse genome (GRCm38: https://www.ncbi.nlm.nih.gov/assembly/GCF_000001635.20/) are available in the NCBI to Gene Expression Omnibus with accession number GSE245266.
