# Supplementary material for: In Situ Formation of Fibronectin‐Enriched Protein Corona on Epigenetic Nanocarrier for Enhanced Synthetic Lethal Therapy
Source: Adv Sci (Weinh). 2024 Mar 14;11(19):2307940. doi: 10.1002/advs.202307940 (PMC11109615; doi:10.1002/advs.202307940)
Supplement: Supplementary file 1 — Supporting Information [file ADVS-11-2307940-s002.pdf]

ADVANCED SCIENCE
Open Access

# Supporting Information

for *Adv. Sci.*, DOI 10.1002/advs.202307940

In Situ Formation of Fibronectin-Enriched Protein Corona on Epigenetic Nanocarrier for Enhanced Synthetic Lethal Therapy

*Zhangyi Luo, Zhuoya Wan, Pengfei Ren, Bei Zhang, Yixian Huang, Raymond E. West III, Haozhe Huang, Yuang Chen, Thomas D. Nolin, Wen Xie, Junmei Wang, Song Li* and Jingjing Sun**

# Supplementary Materials

## Supplementary methodological information

### Polymer synthesis and chemical characterization

VD monomer was synthesized according to previous publication [1]. The PVD polymer backbone was synthesized by controlled reversible addition-fragmentation chain transfer (RAFT) polymerization of poly(ethylene glycol) methyl ether methacrylate ($M_n$=950) and VD monomer. Briefly, AIBN (3.6 mg, 0.0223 mmol), 4-Cyano-4-(thiobenzoylthio)pentanoic acid (8 mg, 0.0287 mmol), poly(ethylene glycol) methyl ether methacrylate ($M_n$=950) monomer (800 mg, 0.84 mmol), and VD monomer (680 mg, 2.15 mmol) were added into a Schlenk tube containing 2 mL of tetrahydrofuran. After three freeze-pump-thawing cycles, the tube was placed into an oil bath (80 °C) and the reaction mixture was stirred under the protection of $N_2$. After 20 h, the reaction was quenched and the PVD polymer was obtained by precipitation in ether for 2 times.

The PAZA polymer was obtained by conjugation of AZA to PVD backbone. PVD polymer (60 mg, 0.085 mmol -COOH), EDC (200 mg, 1.04 mmol) and HOBT (80 mg, 0.6 mmol) were dissolved in 15 mL of DMSO with the addition of 200 μL of DIPEA. Then, AZA (80 mg, 0.33 mmol) was added to the solution and stirred at room temperature for 72 h. The PAZA polymer was obtained after dialysis against DMSO/water and lyophilization.

The PCyt and PMan polymers were synthesized similarly as PAZA polymer. AZA (80.59 mg, 0.33 mmol) was replaced by Cytidine (80.26mg, 0.33 mmol) and Mannitol (60.12 mg, 0.33 mmol), respectively.

The structures of these polymers were characterized by NMR and IR.

### Molecular Modeling

**Modeling systems.** The PAZA polymer was constructed to make different residues evenly distributed and the sequence is as the follows: NOE-VD-AZA-OEG-VD-VD-OEG-AZA VD-OEG-VD-VD-AZA-OEG-VD-VD-OEG-AZA-VD-OEG-VD-VD-AZA-OEG-VD-VD-OEG-AZA-VD-OEG-VD-VD-CVD. Thus, PAZA polymer consists of five residue types, which are NOE (N-terminal OEG), VD, AZA, OEG, and CVD (C-terminal VD). Of note, although the numbers of OEG, VD and AZA residues are known from experiment, the exact sequence of the

polymer is unclear. The sequence of PVD is similar to that PAZA except that AZA residue was replaced by VD. Each residue has hydrophilic and hydrophobic moieties, with the hydrophobic moieties tend to be buried inside the micelle. The hydrophobic moieties are colored in red, while the hydrophilic ones in blue in Figures S1. The five residues and BMN-673 were described by the GAFF2[2]. Specifically, we performed *ab initio* calculations at the B3LYP/6-31G*//HF/6-31G* level using Gaussian 16 package[3] to obtain electrostatic potentials (ESP) to derive partial charges, and then applied the Antechamber software package[4] implemented in AMBER 18 software package[5] to generate residue topologies. As there is no experimentally determined structures for PAZA and PVD, we generated an initial structure which has an extended conformation.

**Molecular dynamics simulations.** We applied a series of sequential molecular dynamics (MD) simulations to obtain the micelle structure formed by a single copy of PAZA or PVD polymer. In Step 1, the extended conformation of the polymer was collapsed in explicit water during the 100 nanoseconds (ns) simulation. In Step 2, starting from the last snapshot of Step 1, a Generalized Born molecular dynamics (GBMD) simulation was performed to exclude the trapped water molecules and accelerate micelle structure formation. In Step 3, we solvated last snapshot from Step 2 in a rectangle water box which has dimension of about 70 × 70 × 80 Å, and performed 300-ns MD simulations to sample an isothermal–isobaric (NPT) ensemble of the polymer for the followed free energy analysis. 400 MD snapshots were collected from the last 200 ns for post-analysis. For the sake of binding free energy calculation, we also performed NPT simulations for BMN-673 solvated in a cubic water box with dimension of about 45 Å. 1000 MD snapshots were evenly collected for the MM-PBSA-WSAS free energy calculation.

To simulate how BMN drugs interact with PAZA or PVD polymers, we placed 8 copies of BMN molecules at the vertices of a cube with edge length of 50 Å, and placed the polymer structure obtained in Step 3 in the center of the cube. Of note, the drug molecules were initially far away from the polymer and had no direct contact with the polymer. The whole system was soaked in a rectangle water box with the dimension of about 80 × 80 × 80 Å. We then performed 300-ns swarm MD simulations and collected 400 snapshots from the last 200 ns for post-analysis.

The values of the key parameters controlling the GBMD and explicit water MD simulation are listed as the follows: the time step of integrating the Newton's equation of motion – 2

femtoseconds, desired temperature – 298 K, desired pressure for NTP simulation – 1 bar. All MD simulations were performed using the pmemd.cuda program in AMBER 18[5]. Due to highly dynamic attribute of the systems, we calculated radius of gyration (RoG) of the polymer and polymer-drug systems to monitor the micelle structure formation and drug-polymer interactions. Additionally, we calculated the root-mean-square deviation (RMSD) of the heavy atoms to describe the dynamics of the polymer and polymer-drug complex. For each MD trajectory, the snapshot which had the smallest RMSD to the average structure was selected as the representative conformation.

**Free energy analysis.** We applied an internal program to calculate the MM-PBSA-WSAS free energies of the drug, the polymer, and the complex formed by the polymer and 8 drug molecules using the MD snapshots collected in the sampling phase of the NPT simulations. For a molecular or molecular system, the polar solvation free energy, $\boldsymbol{G_{sol}^{PB}}$ , was calculated using the Delphi 95 software[6,7], using the interior and exterior dielectric constants of 1.0 and 80.0, respectively. The nonpolar solvation free energy, $\boldsymbol{G_{sol}^{SA}}$ , was estimated using the solvent accessible surface area (SAS) as detailed elsewhere[8,9]. The conformational entropy of the system, TS, was predicted using WSAS, a weighted solvent-accessible surface area approach[10]. Due to the dramatic conformational change upon drug binding and the highly dynamic nature of the polymer, the "three-trajectories" protocol, for which the free energies were calculated using three individual MD trajectories sampled for the drug, the polymer, and the complex, is more adequate than the commonly used "one-trajectory" protocol. The MM-PBSA-WSAS binding free energy was calculated using the following formula: $\Delta G_{binding} = G_{complex} - (G_{polymer} + 8 \times G_{BMN})$. Note that the complex contains 8 copies of the BMN molecule. The individual energy terms of MM-PBSA-WSAS free energies of the polymer, the drugs and complexes were listed in Table S_1. The corresponding binding free energy for one copy of BMN is 1/8 of $\Delta G_{binding}$.

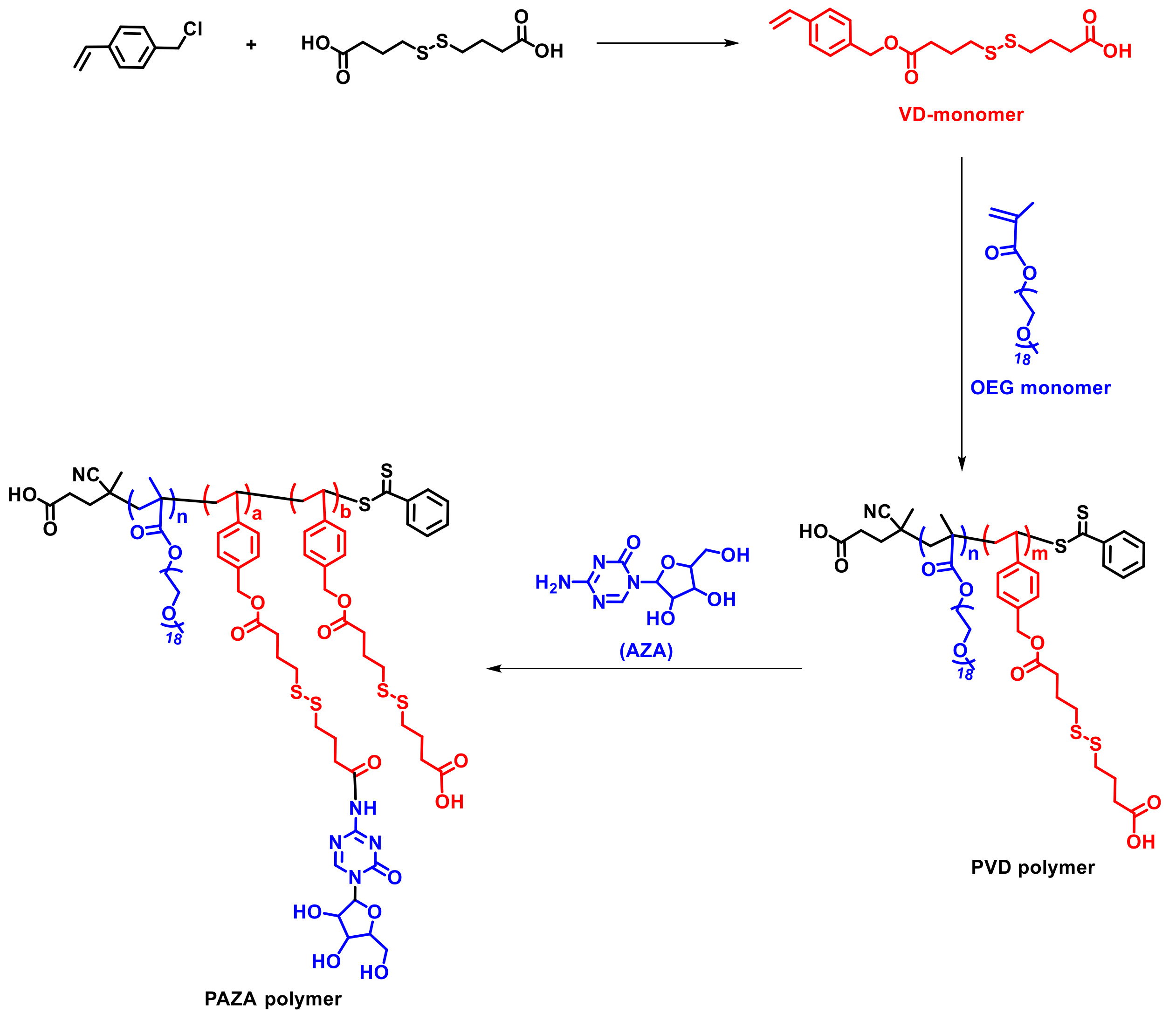


**Figure S1**. Synthesis route of PVD and PAZA polymers by RAFT polymerization and subsequent AZA conjugation.

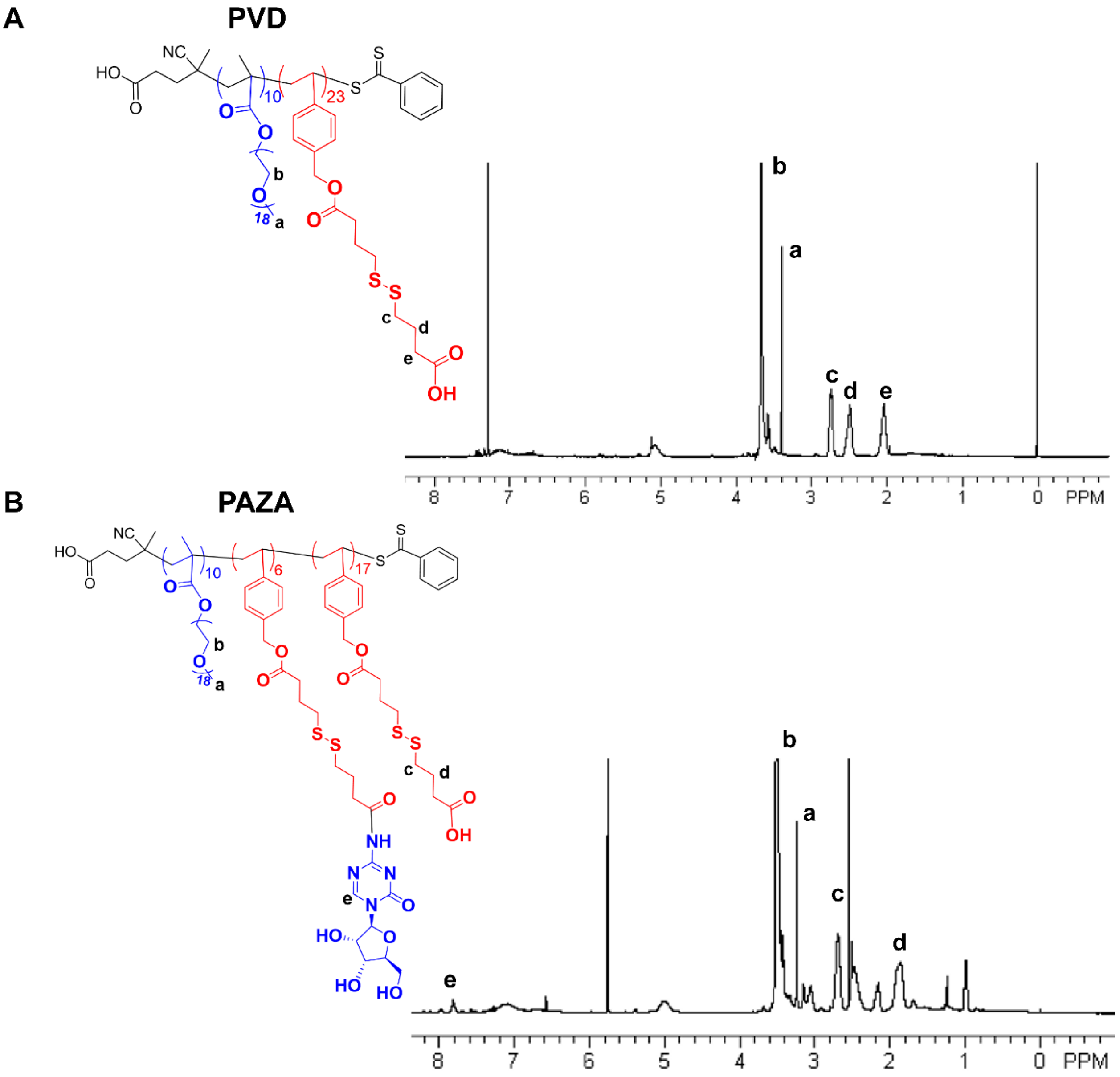


**Figure S2.** NMR characterization of PVD polymer and PAZA polymer. (**A**) PVD polymer. The ratio of PEG unit and VD unit is determined by the integration ratio of peak a to peak c. (**B**) PAZA polymer. The number of poly (ethylene glycol) methyl ether methacrylate and PVD units in the PVD polymer were determined to be 10 and 23, respectively, by comparing the intensities of $I_a$ and $I_c$ in (A). The number of conjugated AZA unit was determined to 6 by comparing the intensities of $I_a$ and $I_e$ in (B).

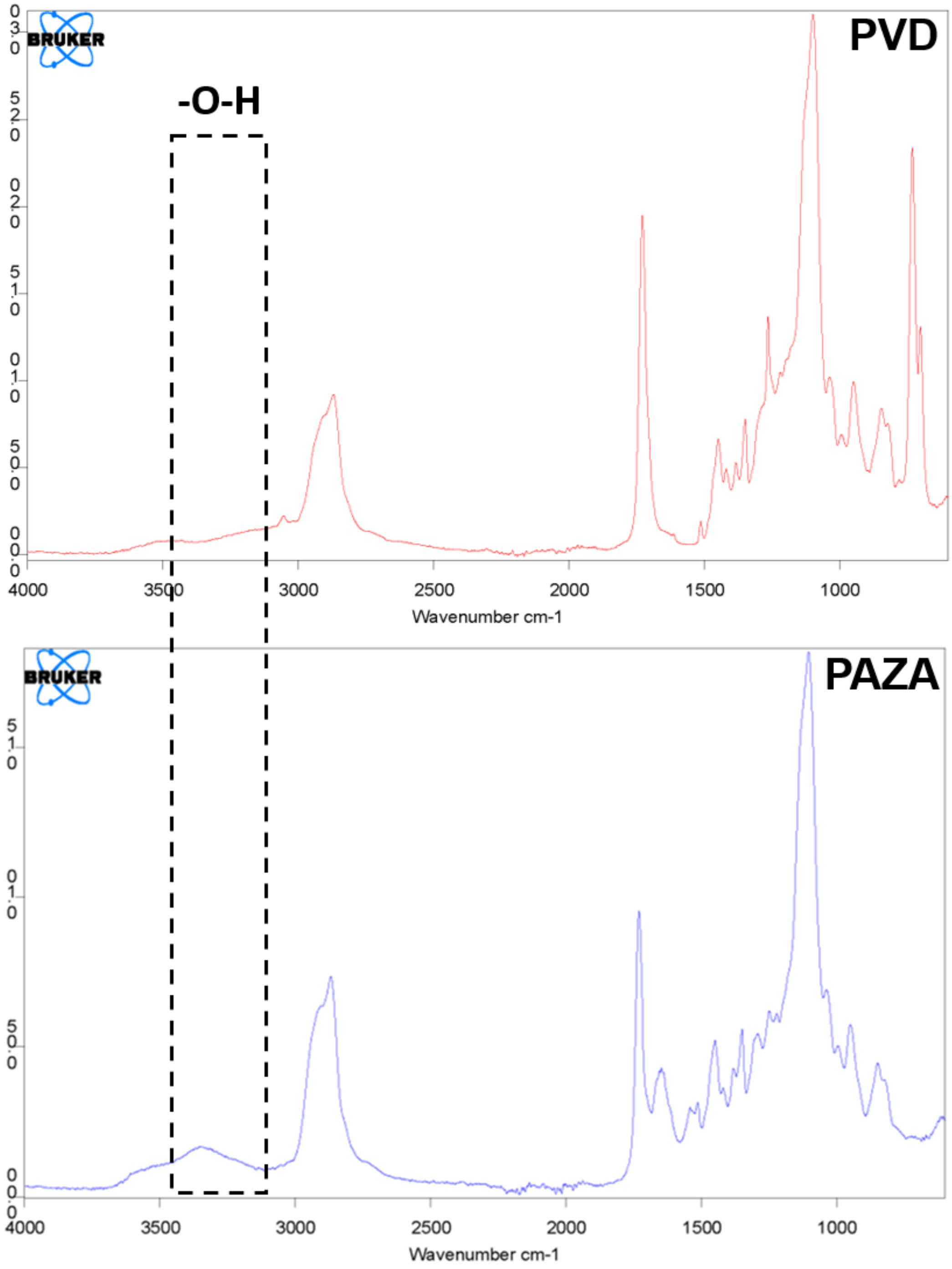


**Figure S3.** IR spectrum characterization of PVD polymer and PAZA polymer. PAZA showed strong broad O-H stretching at 3550-3200.

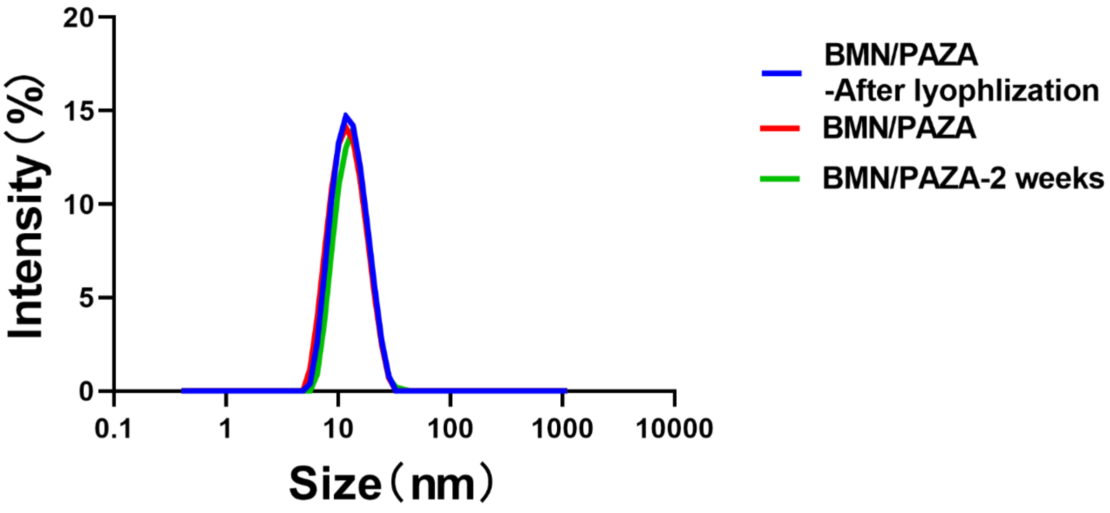


**Figure S4.** Size of BMN/PAZA (red), BMN/PAZA after lyophilization and re-dispersion (blue) and BMN/PAZA under room temperature after 2 weeks (green).

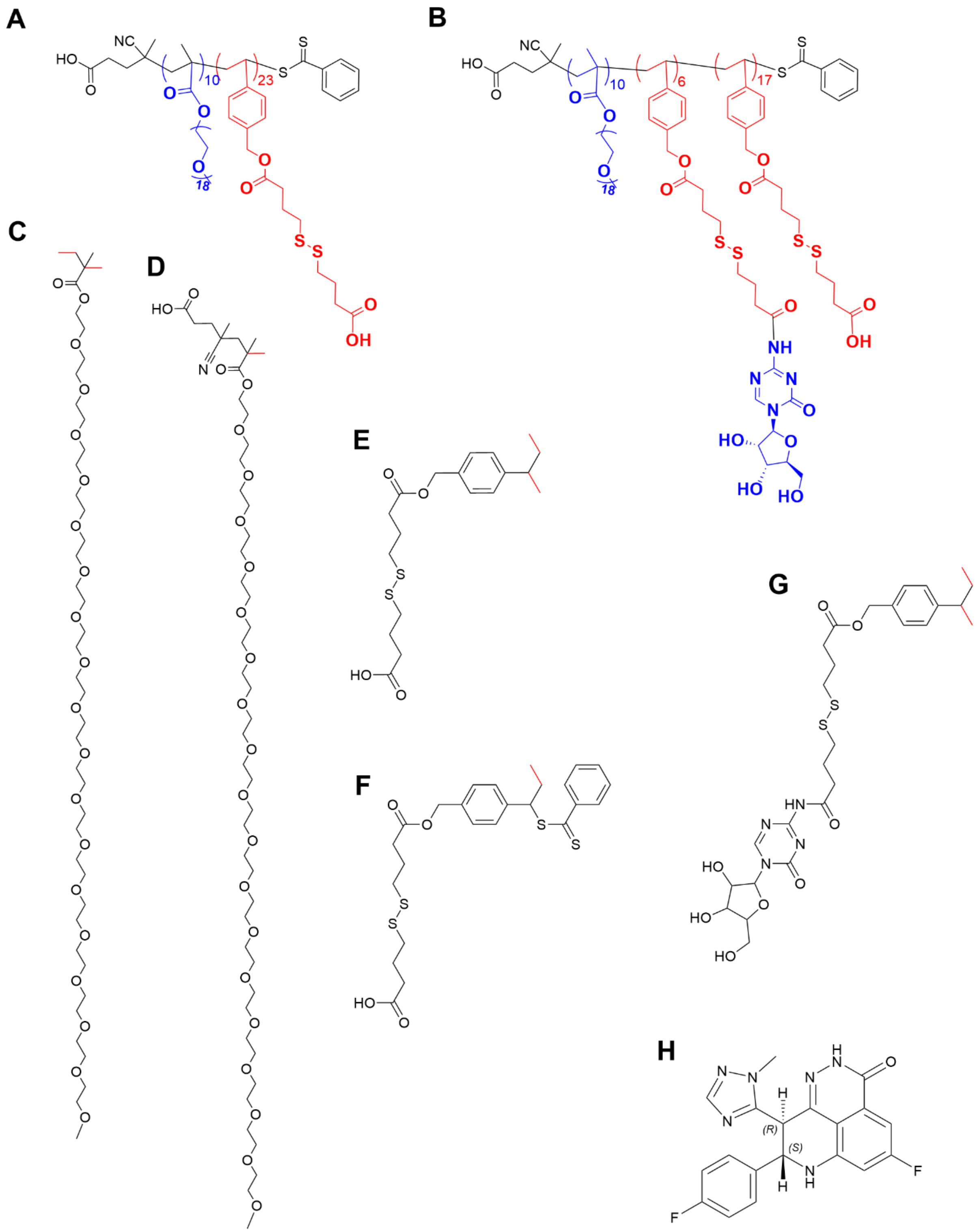


**Figure S5.** The types of residues that used for molecular dynamics simulation of the interaction between BMN and polymer (PVD or PAZA). **(A)** Structure of PVD polymer. **(B)** Structure of PAZA polymer. **(C)** OEG residue. **(D)** "N" -terminal OEG residue. **(E)** VD residue. **(F)** "C" –terminal VD residue. **(G)** AZA residue. **(H)** BMN structure.

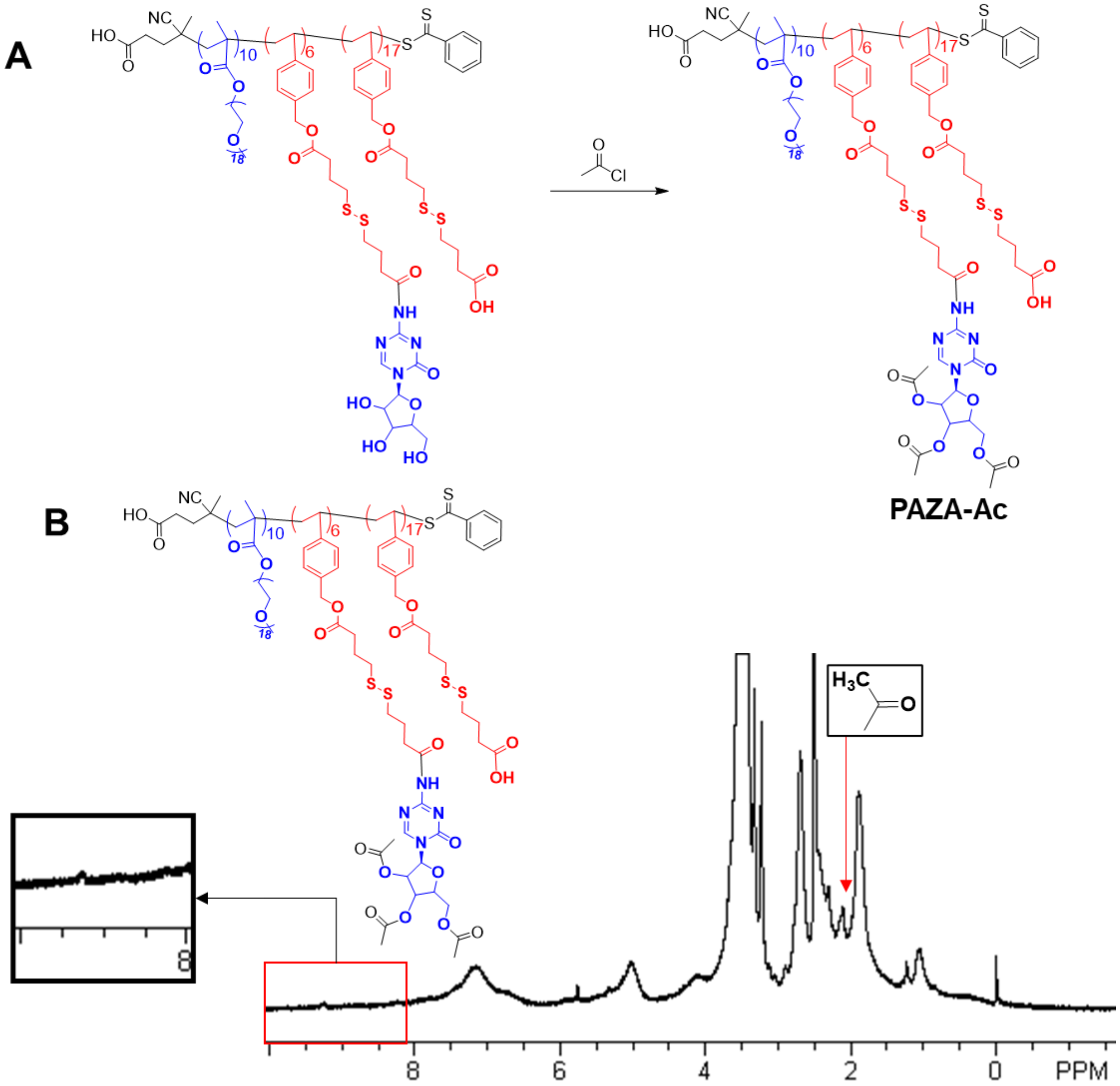


**Figure S6.** (**A**) Synthesis and (**B**)NMR characterization of acetalized PAZA polymer (PAZA-ac).

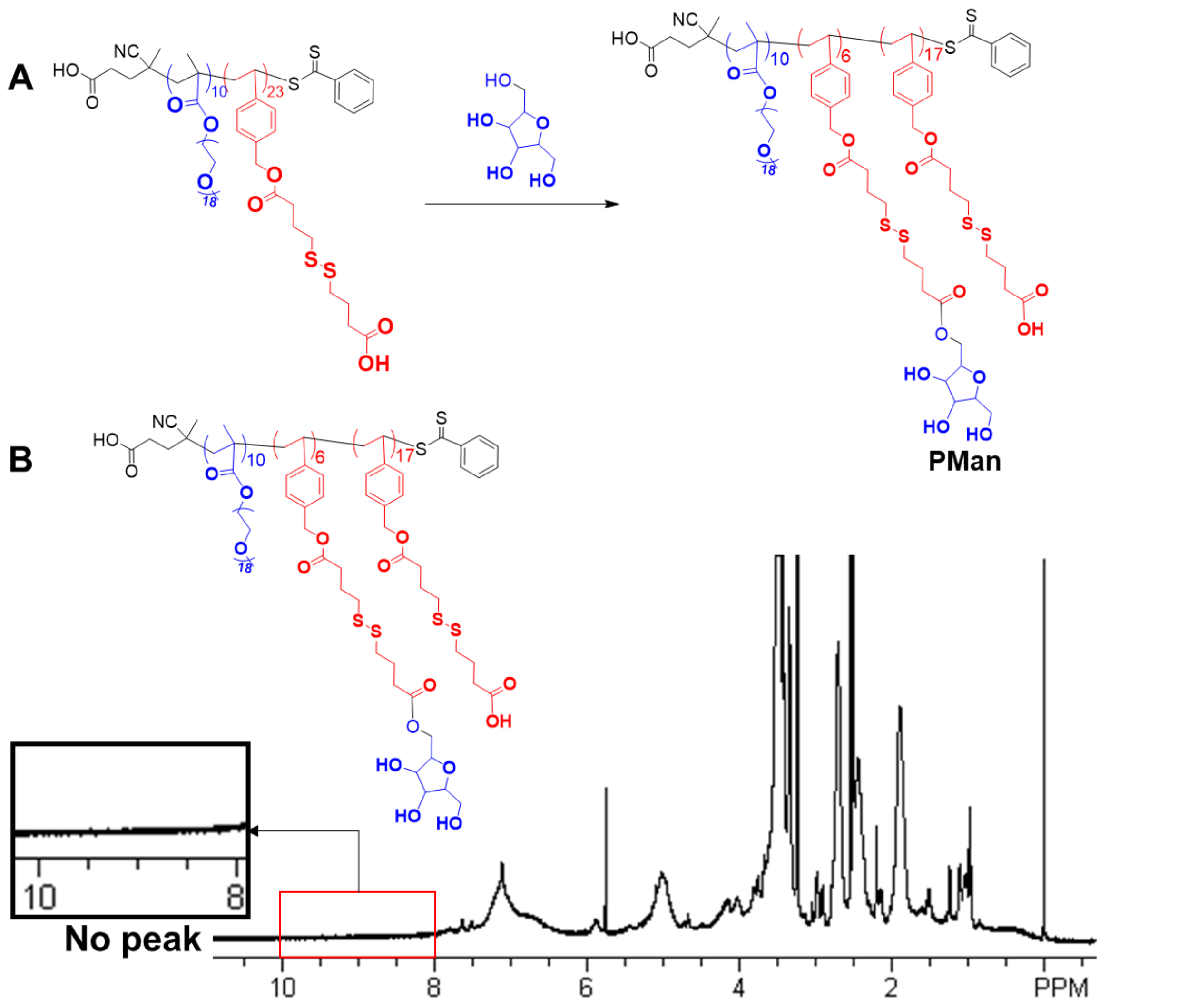


**Figure S7.** (**A**) Synthesis and (**B**) NMR characterization of mannitol conjugated PVD polymer (PMan). There was no pyridine peak shown on NMR.

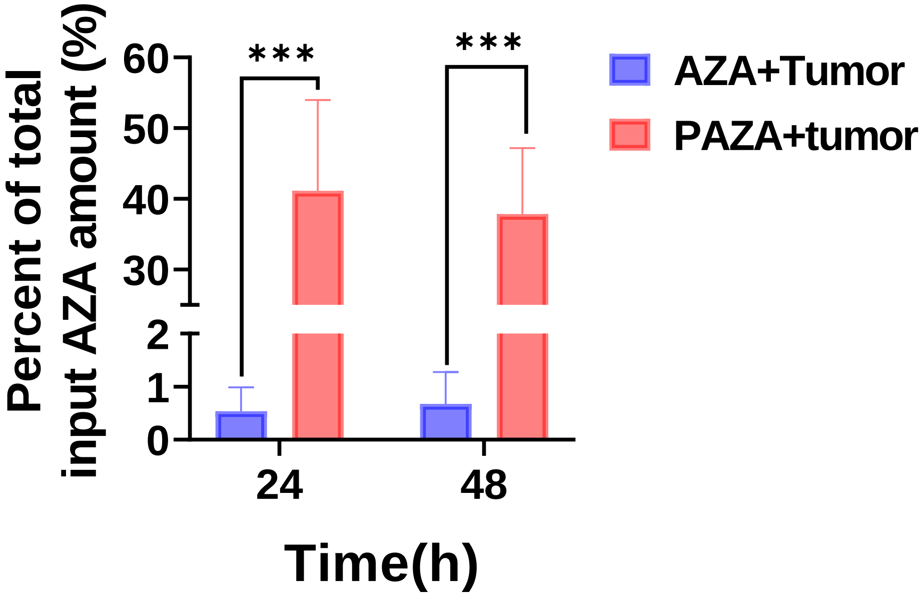


**Figure S8.** The stability of AZA in the tumor lysate. Free AZA and PAZA was incubated with tumor lysate at 24 h and 48 h, the AZA amounts were detected by HPLC. n=3 independent samples, and the data are presented as the mean±s.d. ***p<0.001.

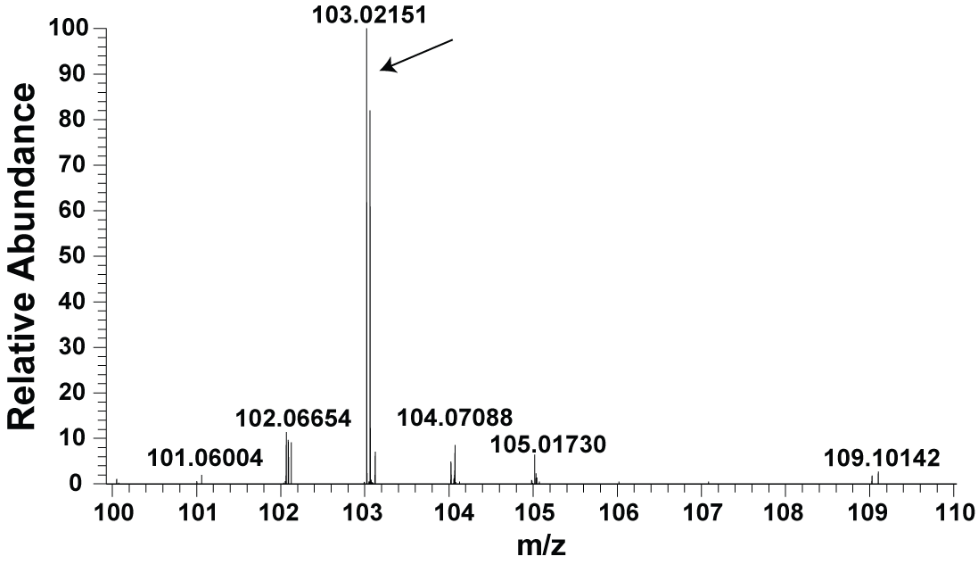


**Figure S9.** Detection of thiolan-2-one (M+1: 103) by ESI-MS.

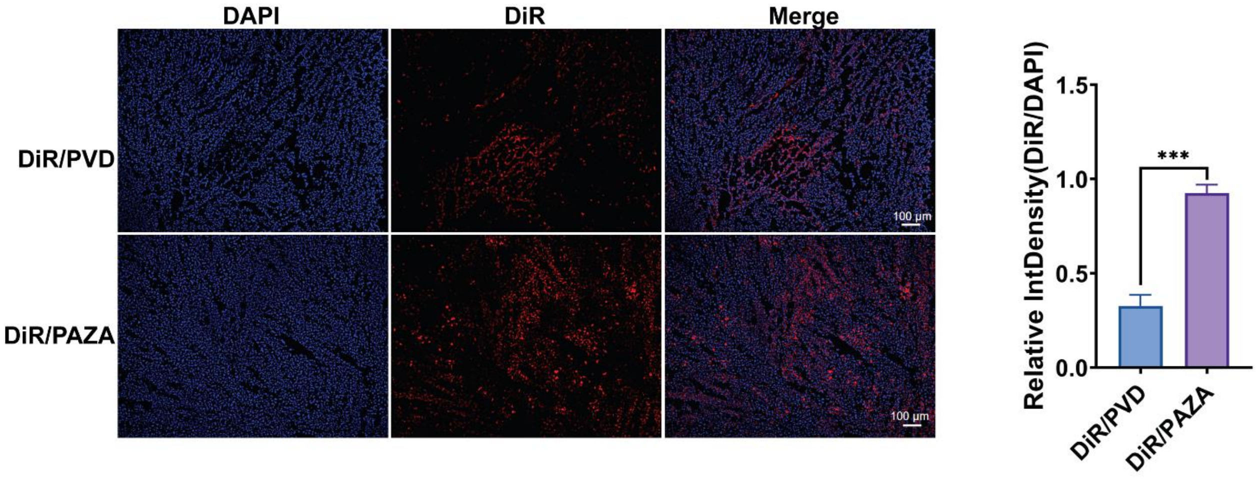


**Figure S10.** Fluorescence images and quantification of frozen tumor core sections at 24 h after treatment with DiR-loaded PVD and PAZA micelles, respectively. (n=3, data are presented as the mean±s.d $*p < 0.05$, $**p < 0.01$, $***p < 0.001$, $****p<0.0001$)

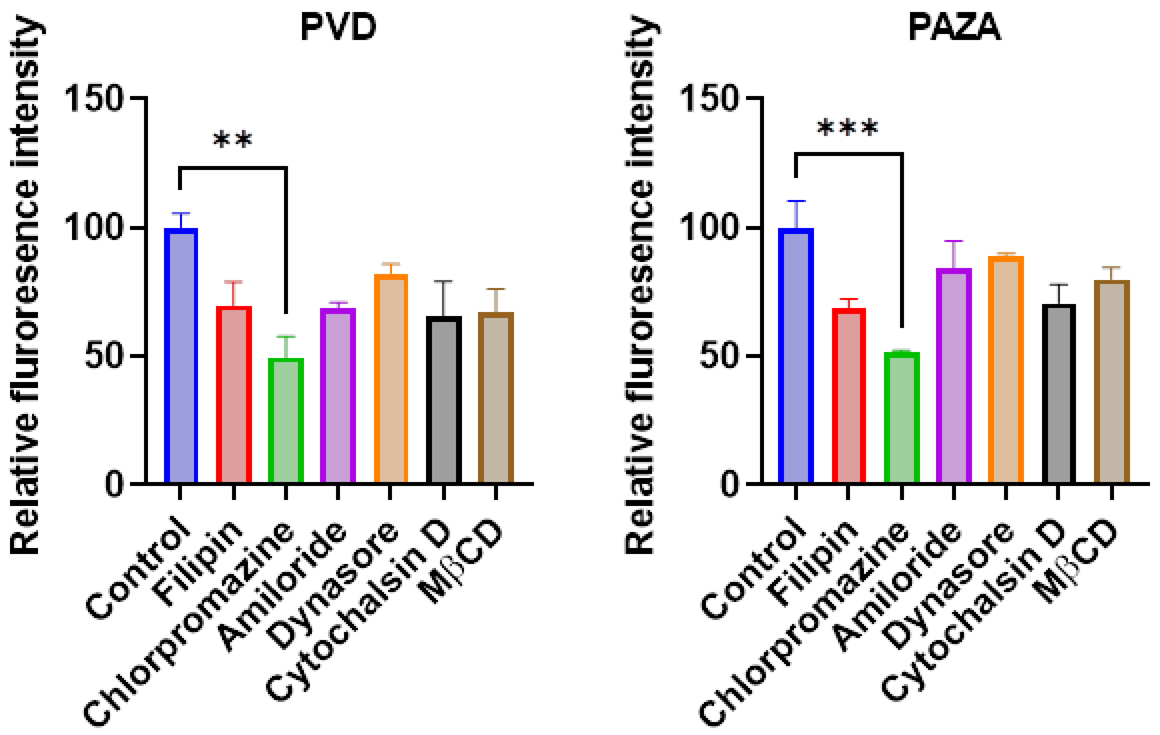


**Figure S11.** Cellular uptake of PVD and PAZA after treated with various inhibitors of endocytic proteins, represented by the quantitative median fluorescence intensity. (n=3, data are presented as the mean±s.d **p < 0.01, ***p<0.001).

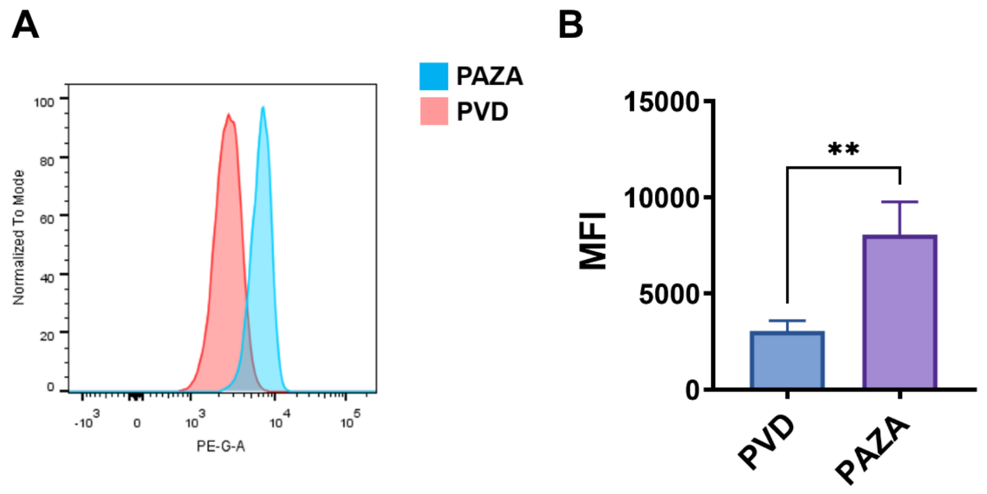


**Figure S12.** Cellular uptake of PAZA and PVD in the lower chamber in a transwell assay. (**A**) The histogram of the Rhodamine fluorescence intensity of lower chamber cell after different carrier treatments. **(B)** The quantitative median fluorescence intensity of the lower chamber cell

after different carrier treatments. n=3, data are presented as the mean±s.d, *p < 0.05, **p < 0.01, ***p < 0.001.

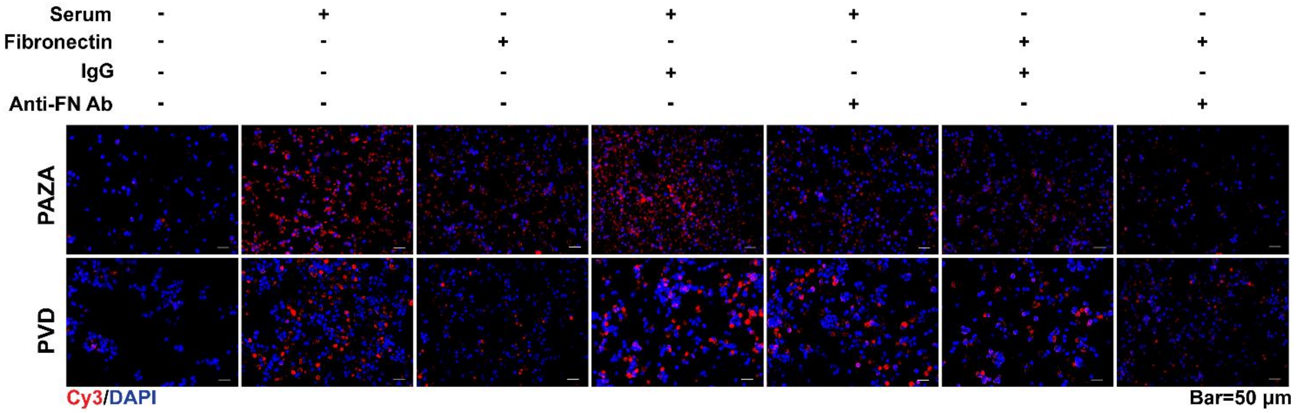


**Figure S13.** Fluorescence images of the cellular uptake of cy3-labelled PAZA and PVD after various treatments. Bar=50μm.

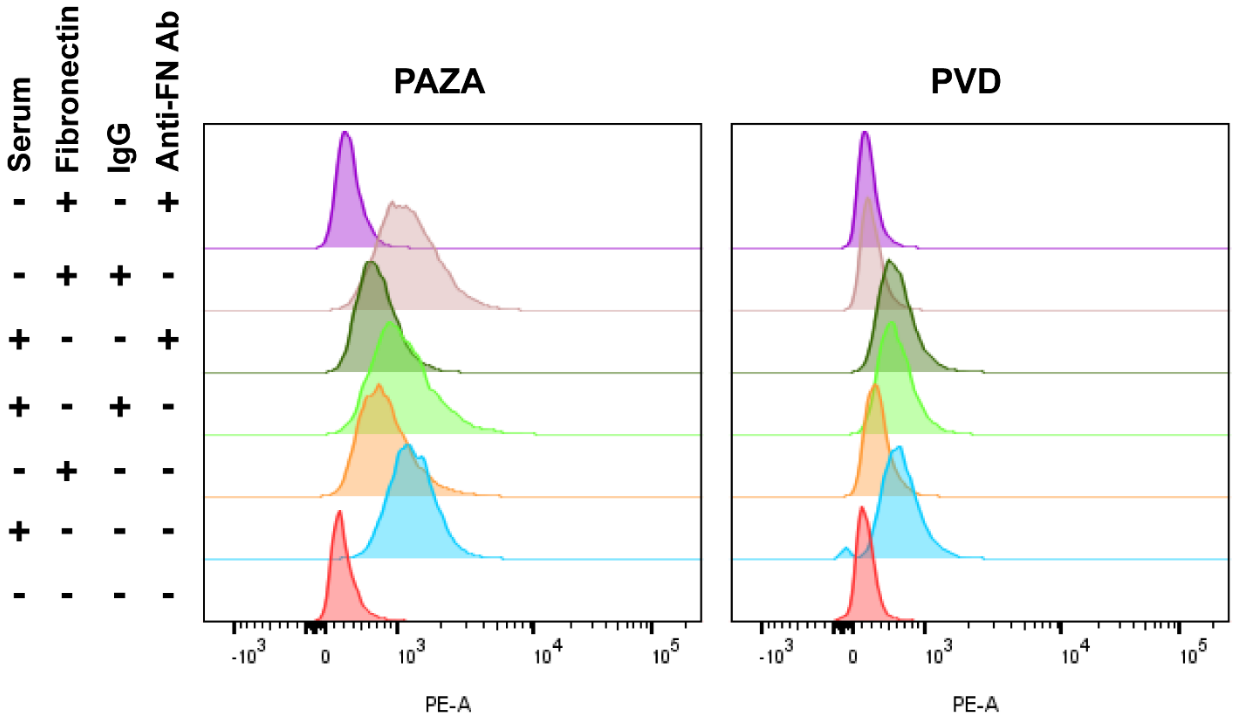


**Figure S14.** Representative histogram of the cellular uptake of cy3-labelled PAZA and PVD after different carrier treatments.

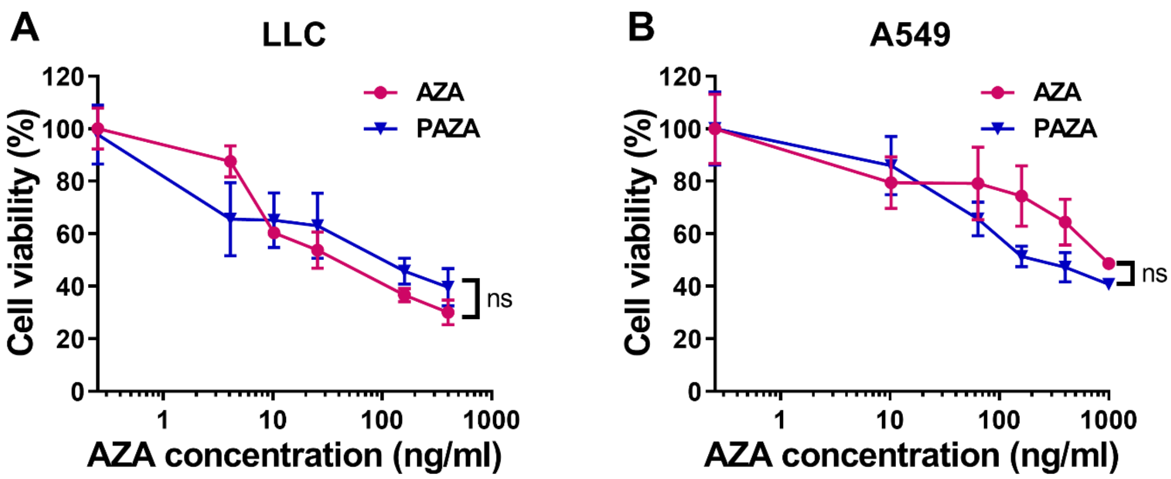


**Figure S15.** MTT cytotoxicity assay of AZA and PAZA on (A) LLC cell line and (B) A549 cell line (n=6).

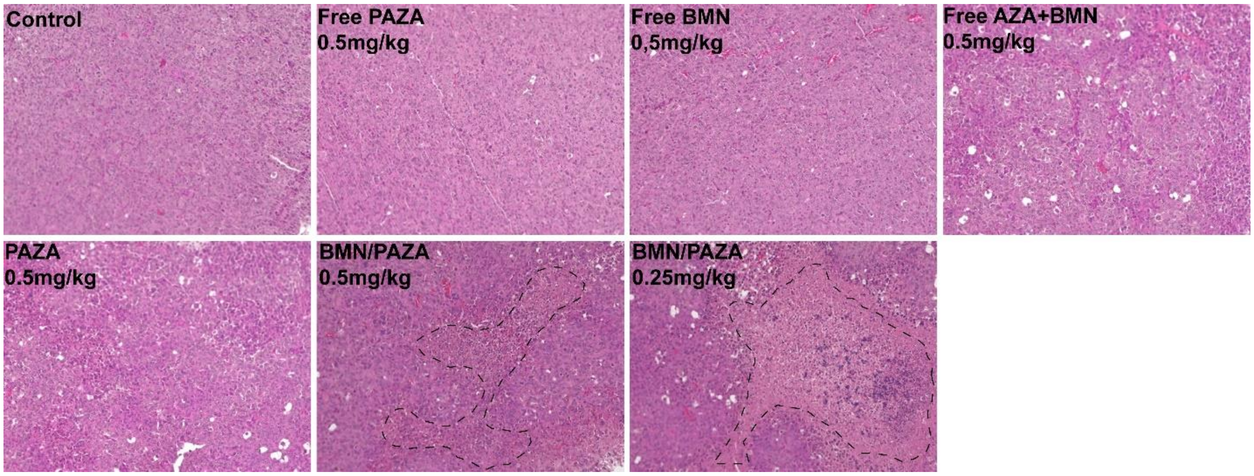


**Figure S16.** H&E staining of tumor tissues from various treatment groups. Necrosis area is defined by dash lines.

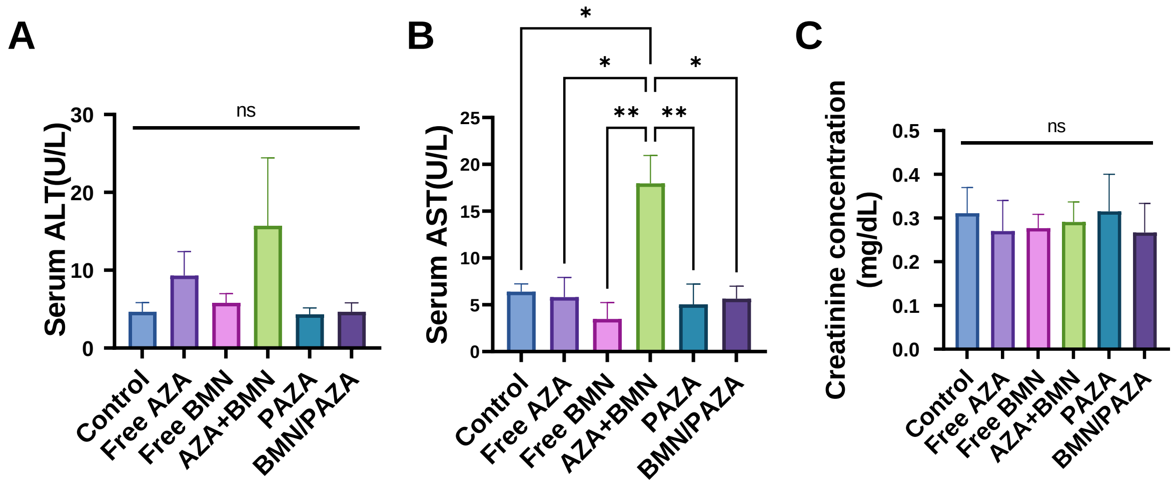


**Figure S17.** Serum level of ALT, AST, and creatinine after various treatments. The dose of both BMN and AZA is 0.5mg/kg. n=3, data are presented as the mean±s.d, *p < 0.05, **p < 0.01, ***p < 0.001.

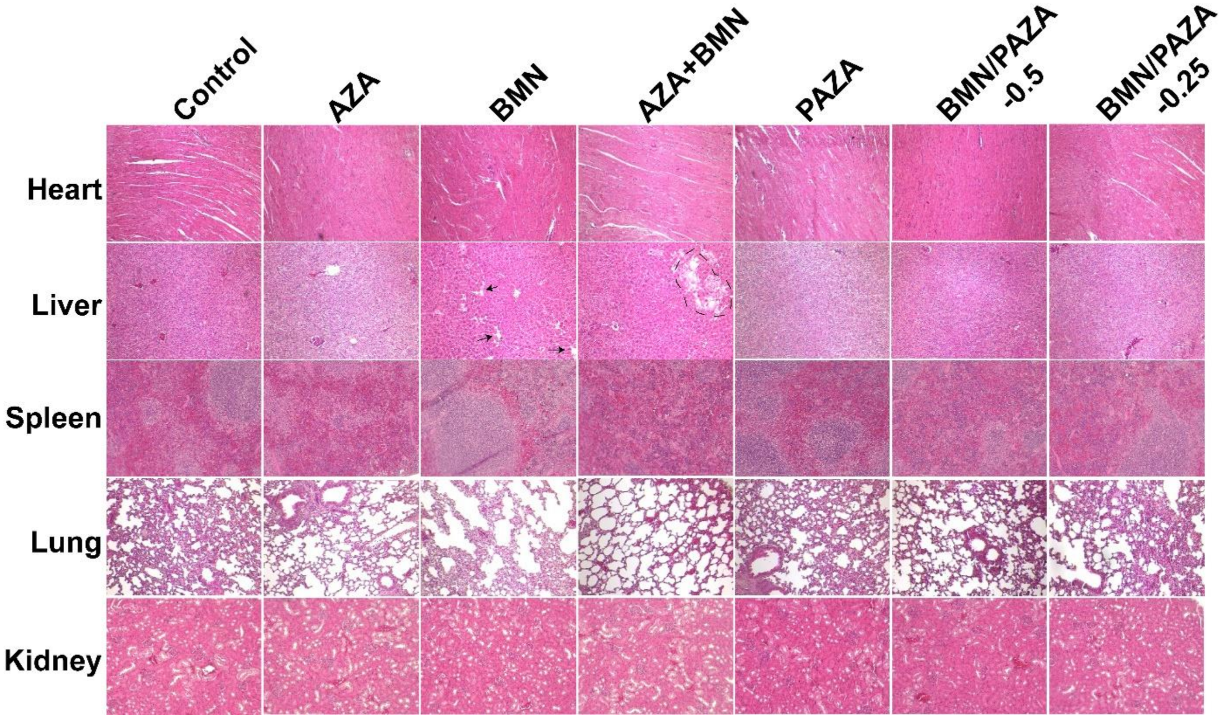


**Figure S18.** H&E staining of major organs after various treatments for 5 times. Arrow and dash line area are possible necrosis areas in liver tissue. The group treated with free BMN or free

BMN and AZA combination showed a little increased cavity in the liver tissue section, while the mice treated with BMN/PAZA showed no significant morphology change in major normal tissues.

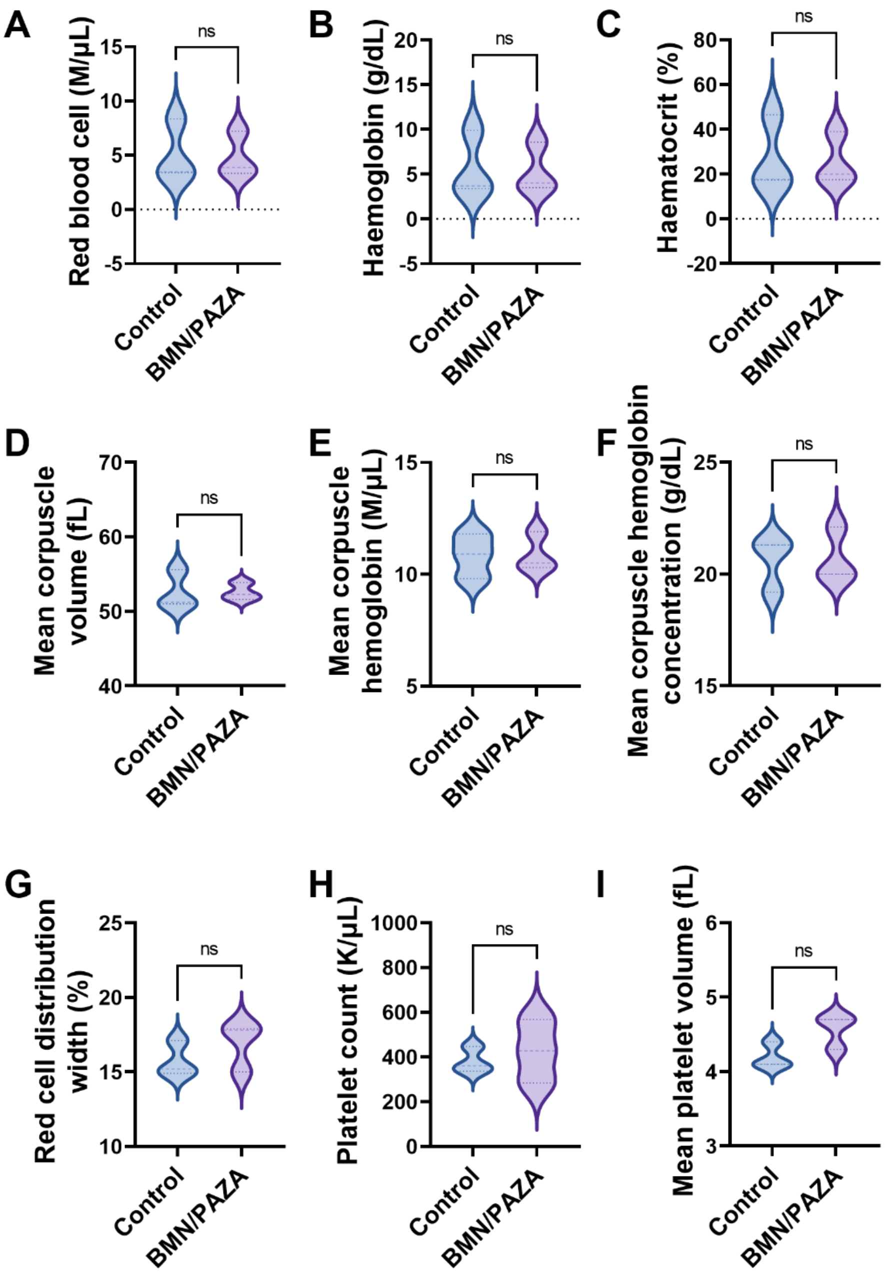


**Figure S19.** Erythrocytes and haem regulation parameters of control group and BMN/PAZA group**.** (A) Red Blood Cells, (B) Hemoglobin, (C) Hematocrit, (D) Mean corpuscle volume, (E) Mean corpuscle hemoglobin, (F) Mean corpuscle hemoglobin concentration, (G) Red cell distribution width, (H) Platelet count, (I) Mean platelet volume. n=3, $*p < 0.05$, $**p < 0.01$, $***p < 0.001$.

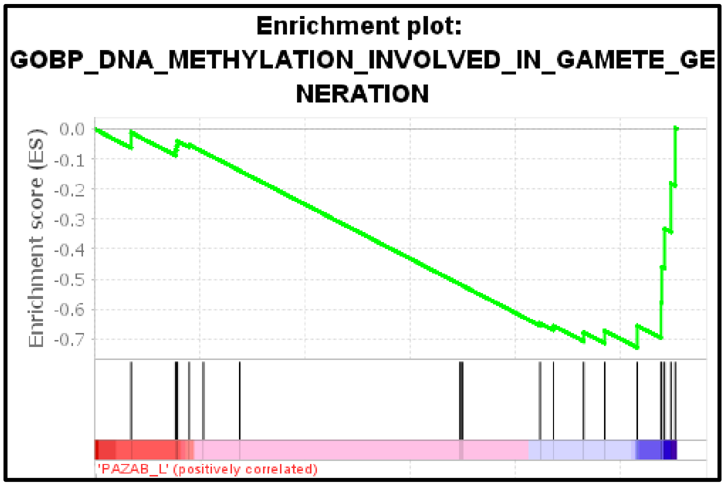


**Figure S20.** Gene Set Enrichment Analysis (GSEA) plot of transcriptional profiles in DNA methylation pathway from tumor tissues of the mice treated with BMN/PAZA versus BMN.

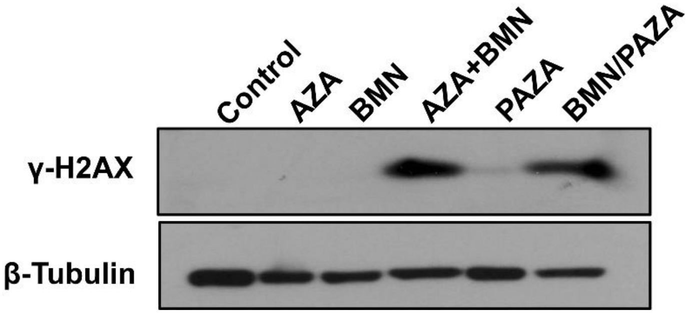


**Figure S21.** γ-H2AX levels in A549 cell line after treatment with AZA, BMN, free AZA plus BMN, PAZA and BMN/PAZA for 48 h, respectively.

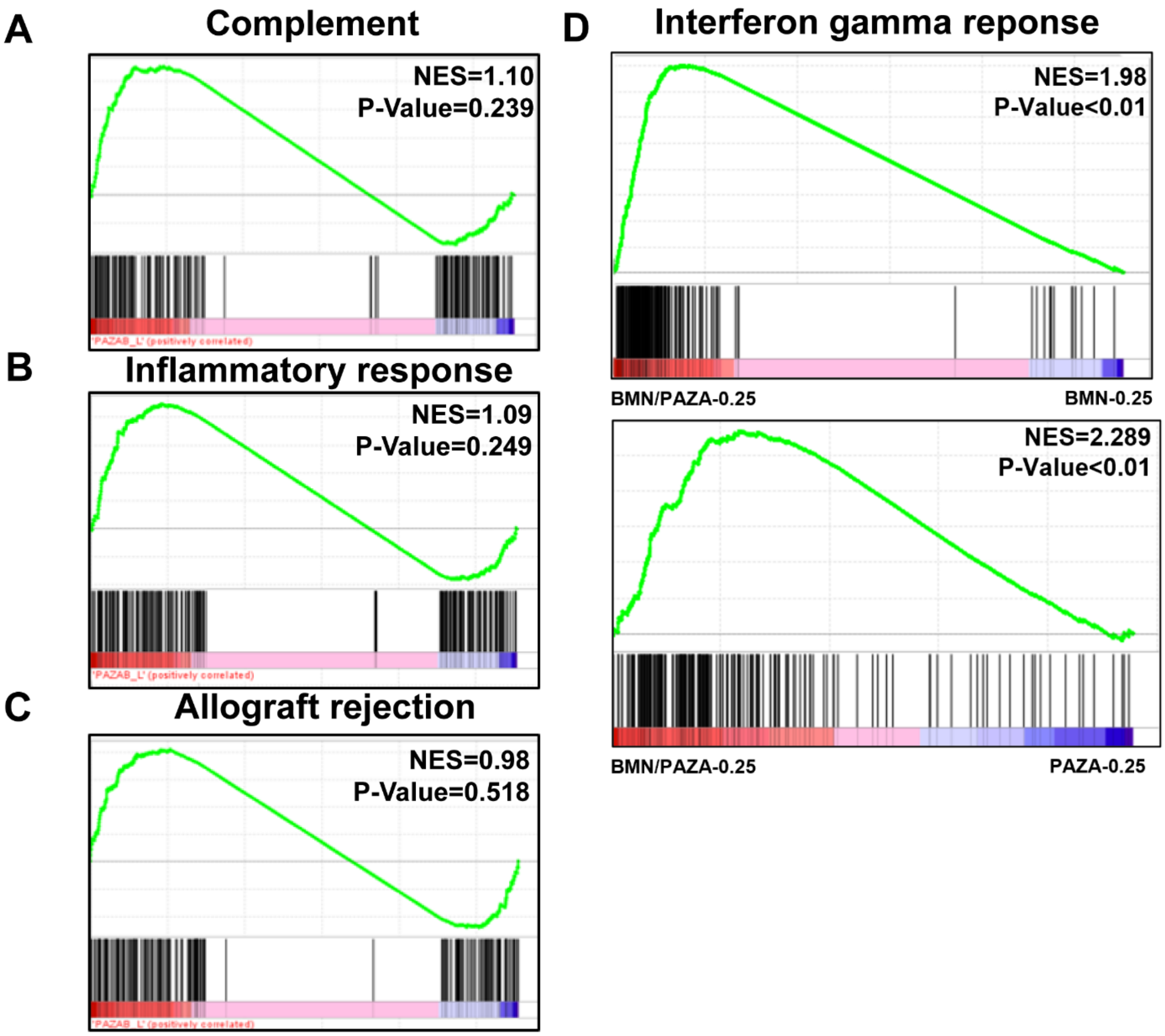


**Figure S22.** Gene Set Enrichment Analysis (GSEA) plot of tumor tissues from BMN/PAZA-0.25 treated mice on various pathway. (A-C) BMN/PAZA-0.25 versus BMN-0.25; (D) BMN/PAZA-0.25 versus BMN-0.25 and BMN/PAZA-0.25 versus PAZA-0.25 (BMN and AZA dosage: 0.25 mg/kg).

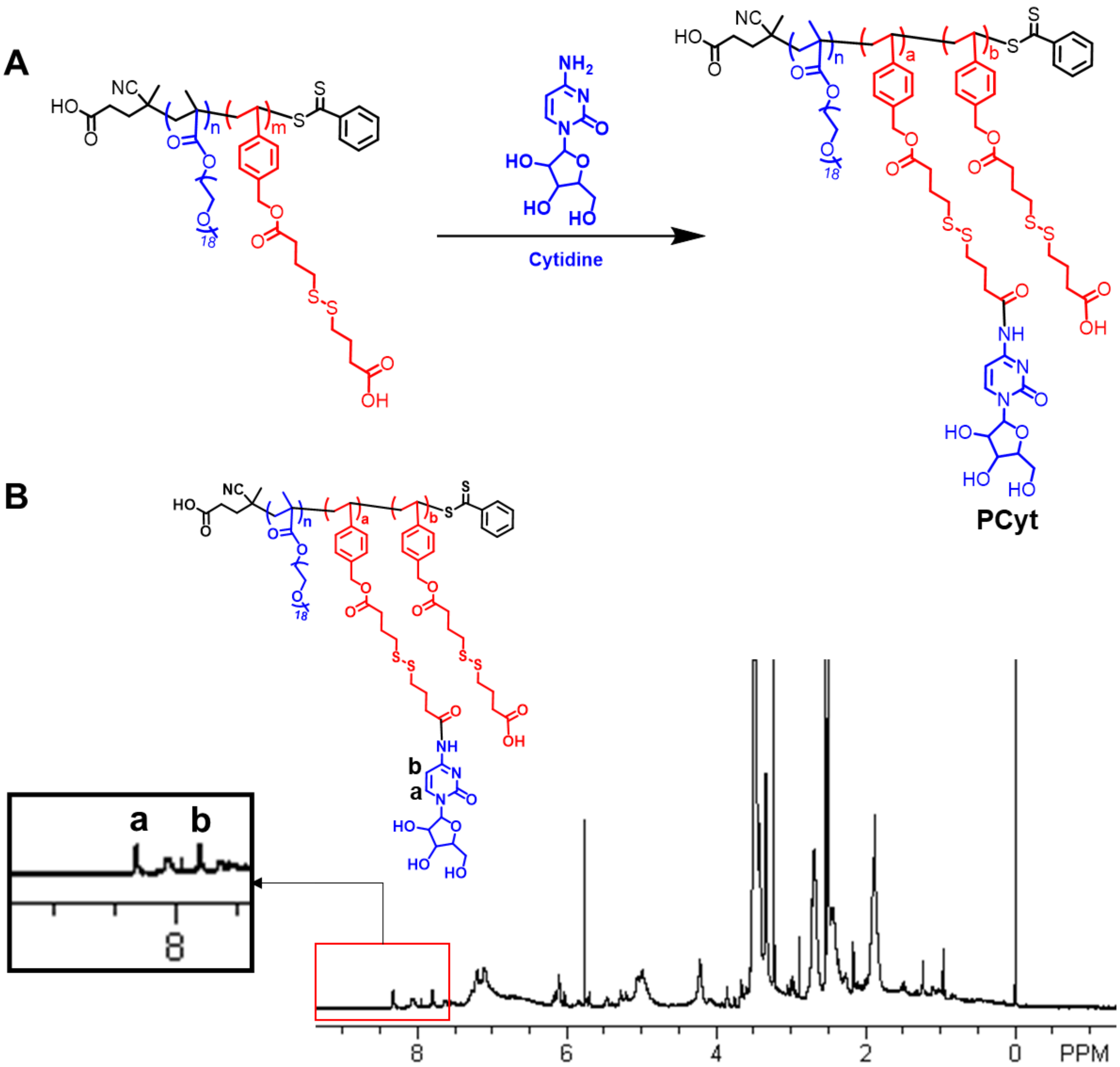


**Figure S23.** (**A**) Synthesis and (**B**) NMR characterization of PCyt polymer by conjugating cytidine to PVD polymer.

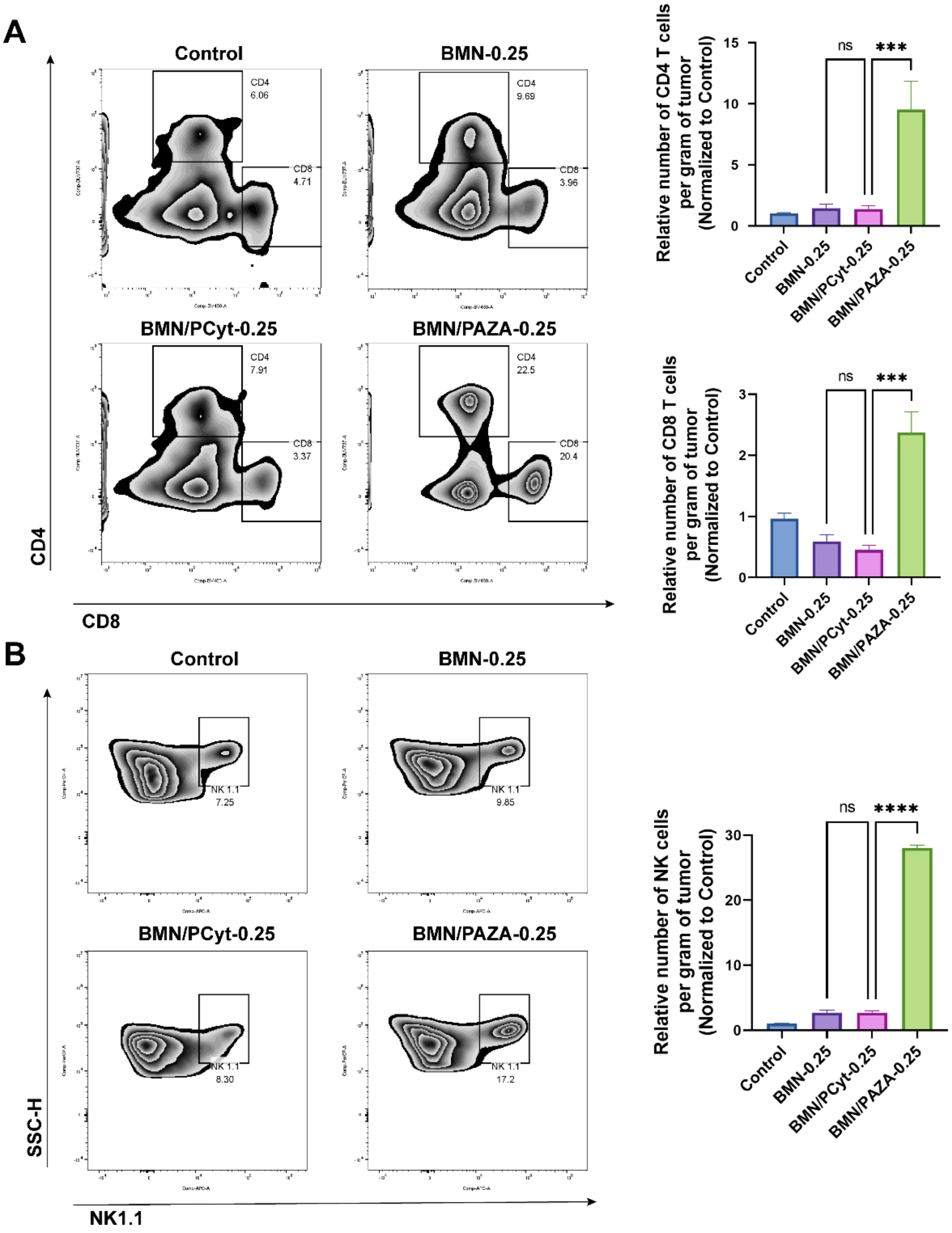


**Figure S24.** Flow cytometry analysis of immune cells, including CD4$^{+}$ T cells (A), CD8$^{+}$ T cells (B) and NK cells (C) in tumor tissues after treatments with BMN, BMN/PCyt-0.25 (0.25 mg/kg of BMN and cytidine), and BMN/PAZA-0.25 (0.25 mg/kg of BMN and AZA). n=3, data are presented as the mean±s.e.m, *p < 0.05, **p < 0.01, ***p < 0.001.

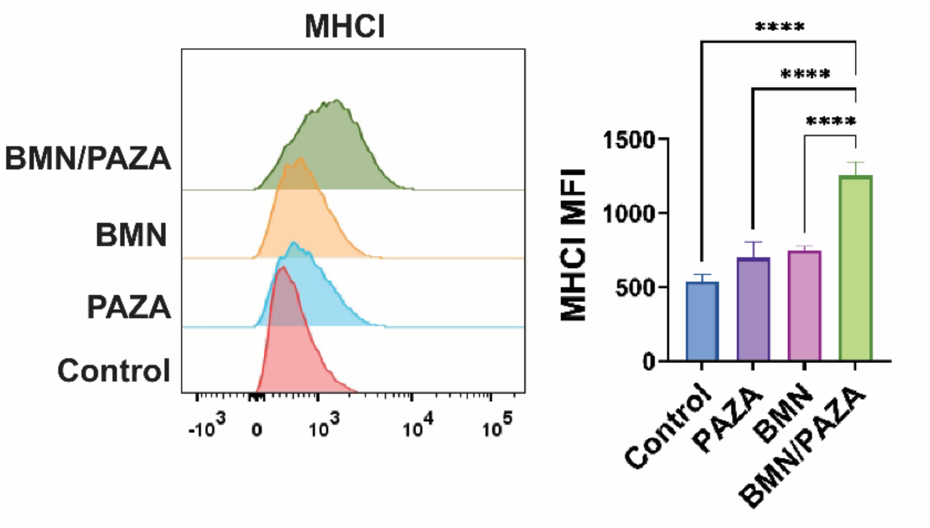


**Figure S25.** Flow cytometry analysis of MHCI on LLC cell after treatment with BMN, PAZA, or BMN/PAZA complex. n=3, data are presented as the mean±s.d, *p < 0.05, **p < 0.01, ***p < 0.001, ****p < 0.0001.

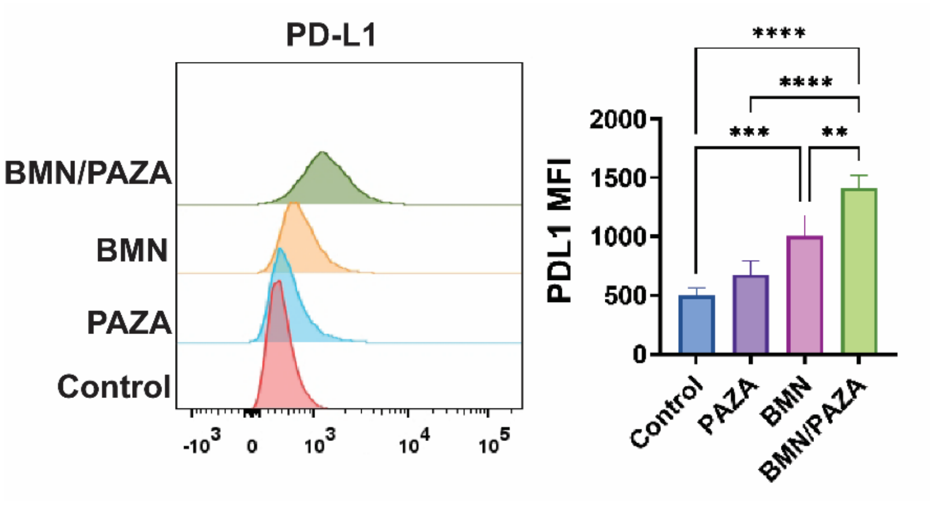


**Figure S26.** Flow cytometry analysis of PD-L1 expression on LLC cell after treatment with BMN, PAZA, or BMN/PAZA complex. n=3. data are presented as the mean±s.d, *p < 0.05, **p < 0.01, ***p < 0.001, ****p < 0.0001.

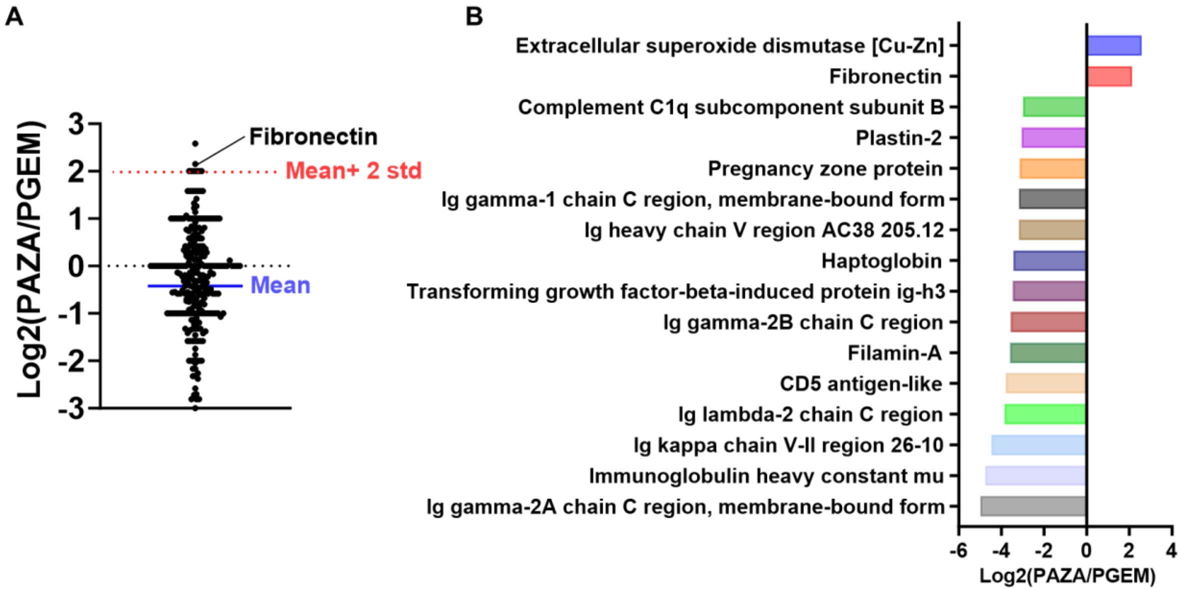


**Figure S27. (A)** Difference of various protein amount in the protein corona on PGEM & PAZA. **(B)** The significantly changed protein corona component of PAZA compared to protein corona on PGEM (> or < two standard deviation)

**Table S1.** List of the free energy terms of seven molecules/molecular systems. All energies are in kcal/mol. $E_{int}$ is the total energy of the bonded terms and T is set to 298.15 K.

| ***System*** | $E_{int}$ | $E_{VDW}$ | $E_{eel}$ | $G^{PB}_{sol}$ | $G^{SA}_{sol}$ | ***TS*** | $G_{MM\text{-}PBSA\text{-}WSAS}$ |
|---|---|---|---|---|---|---|---|
| **BMN** | 47.17 ± 0.14 | 6.70 ±0.06 | -94.61 ±0.07 | -21.96 ±0.03 | 4.27 ±0.00 | 45.33 ±0.01 | -103.76 ±0.11 |
| **PAZA** | 4118.42 ±2.51 | -480.02 ±0.29 | -1272.59 ±3.33 | -1021.10 ± 1.60 | 71.69 ± 0.13 | 2003.95 ±0.78 | -587.55 ±1.52 |
| **PVD** | 3799.64 ±2.05 | -462.40 ±2.05 | 1008.63 ±1.45 | -889.28 ± 1.52 | 70.26 ± 0.06 | 1892.21 ± 0.32 | 1634.64 ± 2.40 |
| **PAZA+8 BMN** | 4490.74 ± 4.61 | -646.34 ± 2.71 | -2123.78 ± 1.81 | -1025.53 ± 1.11 | 71.33 ± 0.14 | 2228.66 ± 0.61 | -1462.23 ± 4.00 |
| **PVD+8 BMN** | 4175.65 ± 3.83 | -603.89 ± 1.87 | 193.50 ± 1.29 | -945.64 ± 1.29 | 72.16 ± 0.10 | 2125.54 ± 0.31 | 766.23 ± 2.96 |

**Table S2.** Logic matrix illustrating the effects of chemical inhibitors we used on different endocytic pathways.

| | Targeted endocytosis pathway | | | |
|---|---|---|---|---|
| | Clathrin-Mediated | Caveolin-Mediated | Macropinocytosis | Phagocytosis |
| **Filipin** | | × | | |
| **Chlorpromazine** | × | | | |
| **Amiloride** | | | × | |
| **Dynasore** | × | | | |
| **Cytochalasin D** | | | | × |
| **MβCD** | | × | | |

**Table S3.** Rank of the abundance of various protein in the protein corona of PAZA,PGEM and PVD

| Abundance Rank | PAZA | PGEM | PVD |
|---|---|---|---|
| 1 | Albumin | Albumin | Albumin |
| 2 | Complement C3 | Complement C3 | Serine protease inhibitor A3K |
| 3 | Serotransferrin | Serotransferrin | Serotransferrin |
| 4 | Serine protease inhibitor A3K | Pregnancy zone protein | Complement C3 |
| 5 | Alpha-1-antitrypsin 1-1 | Murinoglobulin-1 | Alpha-1-antitrypsin 1-1 |
| 6 | Murinoglobulin-1 | Complement factor H | Murinoglobulin-1 |
| 7 | Hemopexin | Ceruloplasmin | Hemopexin |
| 8 | Alpha-1-antitrypsin 1-2 | Ig gamma-2A chain C region, membrane-bound form | Alpha-1-antitrypsin 1-2 |
| 9 | Alpha-1-antitrypsin 1-4 | Apolipoprotein B-100 | Serine protease inhibitor A3M |
| 10 | Ceruloplasmin | Hemopexin | Ceruloplasmin |
| 11 | Serine protease inhibitor A3M | Alpha-1-antitrypsin 1-1 | Alpha-1-antitrypsin 1-4 |
| 12 | Complement factor H | Murinoglobulin-2 | Plasminogen |
| 13 | Vitamin D-binding protein | Serine protease inhibitor A3K | Carboxylesterase 1C |
| 14 | Plasminogen | Plasminogen | Vitamin D-binding protein |
| 15 | Apolipoprotein B-100 | Ig gamma-1 chain C region, membrane-bound form | Apolipoprotein B-100 |
| 16 | Complement C5 | Ig gamma-2B chain C region | Inhibitor of carbonic anhydrase |
| 17 | Carboxylesterase 1C | Alpha-1-antitrypsin 1-4 | Afamin |
| 18 | Complement C4-B | Alpha-1-antitrypsin 1-2 | Complement factor B |
| 19 | Antithrombin-III | Complement C4-B | Antithrombin-III |
| 20 | Apolipoprotein A-I | Haptoglobin | Serine protease inhibitor A3N |
| 21 | Kininogen-1 | Inter alpha-trypsin inhibitor, heavy chain 4 | Complement C4-B |
| 22 | Inhibitor of carbonic anhydrase | Complement C5 | Kininogen-1 |

| | | | |
|---|---|---|---|
| 23 | Inter alpha-trypsin inhibitor, heavy chain 4 | Inter-alpha-trypsin inhibitor heavy chain H1 | Pregnancy zone protein |
| 24 | Isoform LMW of Kininogen-1 | Carboxylesterase 1C | Murinoglobulin-2 |
| 25 | Afamin | Ig gamma-3 chain C region | Gelsolin |
| 26 | Inter-alpha-trypsin inhibitor heavy chain H1 | Vitamin D-binding protein | Isoform LMW of Kininogen-1 |
| 27 | Murinoglobulin-2 | Apolipoprotein A-IV | Inter alpha-trypsin inhibitor, heavy chain 4 |
| 28 | Complement factor B | Inter-alpha-trypsin inhibitor heavy chain H3 | Apolipoprotein A-I |
| 29 | Apolipoprotein A-IV | Hemoglobin subunit beta-1 | Apolipoprotein A-IV |
| 30 | Fibronectin | Serine protease inhibitor A3M | Prothrombin |
| 31 | Gelsolin | Prothrombin | Alpha-2-antiplasmin |
| 32 | Prothrombin | Thrombospondin-1 | Complement factor H |
| 33 | Transthyretin | Complement factor B | Phosphatidylinositol-glycan-specific phospholipase D |
| 34 | Alpha-2-antiplasmin | Gelsolin | Inter-alpha-trypsin inhibitor heavy chain H1 |
| 35 | Serine protease inhibitor A3N | Immunoglobulin kappa constant | Inter-alpha-trypsin inhibitor heavy chain H3 |
| 36 | Inter-alpha-trypsin inhibitor heavy chain H2 | Antithrombin-III | Hemoglobin subunit beta-1 |
| 37 | Phosphatidylinositol-glycan-specific phospholipase D | Inhibitor of carbonic anhydrase | Corticosteroid-binding globulin |
| 38 | Hemoglobin subunit beta-1 | Afamin | Carboxypeptidase N subunit 2 |
| 39 | Pregnancy zone protein | Apolipoprotein A-I | Inter-alpha-trypsin inhibitor heavy chain H2 |
| 40 | Inter-alpha-trypsin inhibitor heavy chain H3 | Kininogen-1 | Alpha-2-HS-glycoprotein |
| 41 | Alpha-2-HS-glycoprotein | Ig alpha chain C region | Plasma kallikrein |
| 42 | Apolipoprotein E | Plasma protease C1 inhibitor | Apolipoprotein E |
| 43 | Complement factor I | Hemoglobin subunit alpha | Hemoglobin subunit beta-2 |
| 44 | Corticosteroid-binding globulin | Heparin cofactor 2 | Complement C5 |
| 45 | Plasma kallikrein | Phosphatidylinositol-glycan-specific phospholipase D | Complement component C8 beta chain |
| 46 | Heparin cofactor 2 | Inter-alpha-trypsin inhibitor heavy chain H2 | Leukemia inhibitory factor receptor |
| 47 | Insulin-like growth factor-binding protein complex acid labile subunit | Plasma kallikrein | H-2 class I histocompatibility antigen, Q10 alpha chain |
| 48 | Carboxypeptidase N catalytic chain | Serine protease inhibitor A3N | Heparin cofactor 2 |
| 49 | Leukemia inhibitory factor receptor | Sulfhydryl oxidase 1 | Sulfhydryl oxidase 1 |
| 50 | Plasma protease C1 inhibitor | Complement component C9 | Transferrin receptor protein 1 |

**Table S4.** IC50 of BMN, PAZA carrier, free drug combination, and BMN/PAZA combination on LLC and A549 cell line.

| IC50(ng/mL) | BMN | AZA+BMN | PAZA | BMN/PAZA |
|---|---|---|---|---|
| **LLC** | 325.8 | 146.2 | 4972 | 55.96 |
| **A549** | 814.2 | 120.9 | 108992 | 124 |

**Tables. S5**. Enriched genes in Gene Set Enrichment Analysis for DNA Methylation pathway

| SYMBOL | RANK IN GENE LIST | RANK METRIC SCORE | RUNNING ES | CORE ENRICHMENT |
|---|---|---|---|---|
| TDRD5 | 46485 | -0.632 | -0.6769 | Yes |
| DNMT3L | 48480 | -0.868 | -0.6719 | Yes |
| TDRD12 | 51573 | -1.528 | -0.6553 | Yes |
| PIWIL4 | 53832 | -2.496 | -0.5778 | Yes |
| TDRD9 | 53904 | -2.539 | -0.4588 | Yes |
| ASZ1 | 54160 | -2.773 | -0.3319 | Yes |
| MORC1 | 54783 | -3.408 | -0.1817 | Yes |
| TDRKH | 55178 | -4.048 | 0.0031 | Yes |

**Tables. S6**. Top 50 enriched genes in Gene Set Enrichment Analysis for DNA repair pathway

| SYMBOL | RANK IN GENE LIST | RANK METRIC SCORE | RUNNING ES | CORE ENRICHMENT |
|---|---|---|---|---|
| FEN1 | 54011 | -2.108 | -0.494 | Yes |
| POLR1C | 54039 | -2.129 | -0.4861 | Yes |
| VPS28 | 54058 | -2.15 | -0.478 | Yes |
| RRM2B | 54080 | -2.17 | -0.4699 | Yes |
| GMPR2 | 54114 | -2.208 | -0.4618 | Yes |
| COX17 | 54129 | -2.22 | -0.4533 | Yes |
| RAE1 | 54133 | -2.227 | -0.4447 | Yes |
| POLB | 54150 | -2.249 | -0.4361 | Yes |
| GUK1 | 54176 | -2.278 | -0.4276 | Yes |
| POLR2K | 54198 | -2.298 | -0.419 | Yes |
| CANT1 | 54202 | -2.301 | -0.41 | Yes |
| POLR2G | 54208 | -2.312 | -0.4011 | Yes |
| POLR2J | 54311 | -2.396 | -0.3935 | Yes |
| GTF2H5 | 54360 | -2.423 | -0.3849 | Yes |
| CLP1 | 54386 | -2.453 | -0.3757 | Yes |
| EIF1B | 54387 | -2.454 | -0.3661 | Yes |
| NME1 | 54389 | -2.457 | -0.3564 | Yes |
| POLR2H | 54394 | -2.458 | -0.3469 | Yes |
| ADRM1 | 54396 | -2.461 | -0.3372 | Yes |
| GTF2A2 | 54410 | -2.47 | -0.3278 | Yes |
| SNAPC4 | 54413 | -2.484 | -0.3181 | Yes |
| RBX1 | 54429 | -2.505 | -0.3085 | Yes |
| TK2 | 54447 | -2.533 | -0.2989 | Yes |
| TAF1C | 54449 | -2.535 | -0.289 | Yes |
| RFC3 | 54512 | -2.611 | -0.2798 | Yes |
| TAF10 | 54514 | -2.613 | -0.2696 | Yes |
| MPG | 54540 | -2.657 | -0.2596 | Yes |
| POLR2E | 54554 | -2.675 | -0.2494 | Yes |
| POLD1 | 54568 | -2.704 | -0.239 | Yes |
| POLR2C | 54589 | -2.743 | -0.2286 | Yes |
| MRPL40 | 54602 | -2.765 | -0.218 | Yes |
| ERCC4 | 54611 | -2.787 | -0.2072 | Yes |
| VPS37D | 54622 | -2.796 | -0.1964 | Yes |
| RPA3 | 54631 | -2.8 | -0.1856 | Yes |
| DUT | 54657 | -2.832 | -0.1749 | Yes |
| GPX4 | 54678 | -2.867 | -0.164 | Yes |
| APRT | 54730 | -2.976 | -0.1533 | Yes |
| TAF6 | 54739 | -2.986 | -0.1417 | Yes |
| BOLA2 | 54741 | -2.986 | -0.13 | Yes |
| BRF2 | 54749 | -2.998 | -0.1184 | Yes |
| POLE4 | 54775 | -3.048 | -0.1068 | Yes |
| NT5C | 54810 | -3.095 | -0.0953 | Yes |
| POLR2F | 54814 | -3.097 | -0.0832 | Yes |
| POLD4 | 54833 | -3.121 | -0.0713 | Yes |
| TYMS | 54843 | -3.144 | -0.0591 | Yes |
| POLL | 54906 | -3.251 | -0.0475 | Yes |
| MPC2 | 54937 | -3.303 | -0.0351 | Yes |
| POLR2I | 55008 | -3.471 | -0.0227 | Yes |
| ERCC1 | 55064 | -3.616 | -0.0095 | Yes |
| NME3 | 55104 | -3.732 | 0.0044 | Yes |

**Tables. S7**. Top 50 enriched genes in Gene Set Enrichment Analysis for Complement pathway

| SYMBOL | RANK IN GENE LIST | RANK METRIC SCORE | RUNNING ES | CORE ENRICHMENT |
|---|---|---|---|---|
| CR2 | 314 | 3.75 | 0.0155 | Yes |
| F7 | 386 | 3.641 | 0.0348 | Yes |
| CASP4 | 694 | 3.192 | 0.0473 | Yes |
| C2 | 767 | 3.111 | 0.0636 | Yes |
| PLSCR1 | 901 | 3.049 | 0.0784 | Yes |
| SIRT6 | 1016 | 2.932 | 0.0929 | Yes |
| MMP12 | 1022 | 2.926 | 0.1094 | Yes |
| CCL5 | 1049 | 2.9 | 0.1253 | Yes |
| IL6 | 1196 | 2.763 | 0.1383 | Yes |
| PRSS36 | 1366 | 2.59 | 0.1498 | Yes |
| DGKG | 1374 | 2.58 | 0.1643 | Yes |
| S100A9 | 1431 | 2.543 | 0.1777 | Yes |
| TFPI2 | 1462 | 2.513 | 0.1913 | Yes |
| HSPA1A | 1616 | 2.428 | 0.2023 | Yes |
| PCSK9 | 1673 | 2.401 | 0.2149 | Yes |
| LAP3 | 1900 | 2.36 | 0.2241 | Yes |
| TNFAIP3 | 1911 | 2.355 | 0.2372 | Yes |
| PSMB9 | 2113 | 2.245 | 0.2463 | Yes |
| CASP1 | 2297 | 2.132 | 0.255 | Yes |
| MMP13 | 2347 | 2.104 | 0.266 | Yes |
| PDGFB | 2410 | 2.068 | 0.2766 | Yes |
| SERPING1 | 2519 | 2.02 | 0.2861 | Yes |
| BRPF3 | 2561 | 2.005 | 0.2966 | Yes |
| CASP7 | 2566 | 2 | 0.3079 | Yes |
| OLR1 | 3072 | 1.782 | 0.3088 | Yes |
| CPM | 3083 | 1.777 | 0.3187 | Yes |
| CD46 | 3265 | 1.703 | 0.325 | Yes |
| CASP9 | 3486 | 1.611 | 0.3301 | Yes |
| IRF1 | 3523 | 1.596 | 0.3385 | Yes |
| CSRP1 | 3530 | 1.591 | 0.3474 | Yes |
| CFH | 3573 | 1.576 | 0.3555 | Yes |
| CFB | 3872 | 1.502 | 0.3586 | Yes |
| CP | 3992 | 1.464 | 0.3648 | Yes |
| MAFF | 4091 | 1.43 | 0.3711 | Yes |
| PLAT | 4175 | 1.412 | 0.3775 | Yes |
| RHOG | 4277 | 1.389 | 0.3836 | Yes |
| L3MBTL4 | 4482 | 1.324 | 0.3874 | Yes |
| PIM1 | 4509 | 1.32 | 0.3943 | Yes |
| SERPINC1 | 4547 | 1.308 | 0.4011 | Yes |
| PLA2G4A | 4657 | 1.275 | 0.4063 | Yes |
| SERPINE1 | 4698 | 1.267 | 0.4127 | Yes |
| IRF2 | 4856 | 1.219 | 0.4168 | Yes |
| LIPA | 5128 | 1.155 | 0.4184 | Yes |
| PLAUR | 5229 | 1.138 | 0.423 | Yes |
| CTSB | 5384 | 1.107 | 0.4265 | Yes |
| ATOX1 | 5521 | 1.076 | 0.4301 | Yes |
| GZMA | 5534 | 1.072 | 0.436 | Yes |
| MMP8 | 5582 | 1.059 | 0.4411 | Yes |
| CASP3 | 5691 | 1.033 | 0.445 | Yes |
| GZMB | 5823 | 1.014 | 0.4483 | Yes |

**Tables. S8**. Top 50 enriched genes in Gene Set Enrichment Analysis for Inflammatory pathway

| SYMBOL | RANK IN GENE LIST | RANK METRIC SCORE | RUNNING ES | CORE ENRICHMENT |
|---|---|---|---|---|
| IL15 | 276 | 3.8 | 0.015 | Yes |
| CD69 | 411 | 3.594 | 0.0314 | Yes |
| HPN | 626 | 3.295 | 0.0449 | Yes |
| PDE4B | 1013 | 2.935 | 0.0533 | Yes |
| CCL5 | 1049 | 2.9 | 0.0679 | Yes |
| BST2 | 1077 | 2.864 | 0.0825 | Yes |
| EBI3 | 1141 | 2.798 | 0.096 | Yes |
| IL6 | 1196 | 2.763 | 0.1096 | Yes |
| SLAMF1 | 1322 | 2.632 | 0.1211 | Yes |
| CD40 | 1345 | 2.606 | 0.1344 | Yes |
| CCL2 | 1435 | 2.54 | 0.1462 | Yes |
| HRH1 | 1441 | 2.534 | 0.1594 | Yes |
| IL7R | 1468 | 2.509 | 0.1721 | Yes |
| FFAR2 | 1567 | 2.453 | 0.1832 | Yes |
| CCL7 | 1938 | 2.348 | 0.1889 | Yes |
| TNFRSF9 | 1987 | 2.319 | 0.2002 | Yes |
| NAMPT | 2149 | 2.209 | 0.2089 | Yes |
| CXCR6 | 2192 | 2.183 | 0.2196 | Yes |
| TNFSF10 | 2453 | 2.051 | 0.2257 | Yes |
| CCL24 | 2533 | 2.015 | 0.2348 | Yes |
| SLC28A2 | 2648 | 1.954 | 0.243 | Yes |
| LY6E | 2841 | 1.863 | 0.2493 | Yes |
| SRI | 2908 | 1.827 | 0.2577 | Yes |
| NFKBIA | 2930 | 1.822 | 0.2669 | Yes |
| OLR1 | 3072 | 1.782 | 0.2737 | Yes |
| GCH1 | 3133 | 1.76 | 0.2819 | Yes |
| TNFSF15 | 3184 | 1.739 | 0.2901 | Yes |
| CXCL11 | 3492 | 1.61 | 0.293 | Yes |
| IRF1 | 3523 | 1.596 | 0.3009 | Yes |
| IL10RA | 3524 | 1.596 | 0.3093 | Yes |
| NMI | 3556 | 1.585 | 0.317 | Yes |
| SELE | 3572 | 1.577 | 0.325 | Yes |
| TAPBP | 3595 | 1.573 | 0.3329 | Yes |
| CCR7 | 3596 | 1.572 | 0.3412 | Yes |
| HAS2 | 3654 | 1.549 | 0.3483 | Yes |
| RHOG | 4277 | 1.389 | 0.3443 | Yes |
| TNFSF9 | 4309 | 1.375 | 0.351 | Yes |
| ITGA5 | 4320 | 1.368 | 0.358 | Yes |
| IL18 | 4518 | 1.315 | 0.3613 | Yes |
| CD82 | 4646 | 1.282 | 0.3658 | Yes |
| SERPINE1 | 4698 | 1.267 | 0.3715 | Yes |
| SCARF1 | 4719 | 1.26 | 0.3777 | Yes |
| PCDH7 | 4749 | 1.249 | 0.3838 | Yes |
| RIPK2 | 4976 | 1.189 | 0.3859 | Yes |
| PLAUR | 5229 | 1.138 | 0.3873 | Yes |
| CXCL10 | 5528 | 1.074 | 0.3876 | Yes |
| HIF1A | 5529 | 1.073 | 0.3932 | Yes |
| NFKB1 | 5991 | 0.975 | 0.39 | Yes |
| KCNMB2 | 6024 | 0.968 | 0.3945 | Yes |
| LCP2 | 6065 | 0.966 | 0.3989 | Yes |

**Tables. S9**. Top 50 enriched genes in Gene Set Enrichment Analysis for Allograft rejection pathway

| SYMBOL | RANK IN GENE LIST | RANK METRIC SCORE | RUNNING ES | CORE ENRICHMENT |
|---|---|---|---|---|
| TGFB2 | 46 | 4.596 | 0.0228 | Yes |
| IL12RB1 | 87 | 4.35 | 0.0445 | Yes |
| CXCR3 | 152 | 4.091 | 0.0644 | Yes |
| IL15 | 276 | 3.8 | 0.0817 | Yes |
| TNF | 621 | 3.303 | 0.0925 | Yes |
| C2 | 767 | 3.111 | 0.1058 | Yes |
| CCL5 | 1049 | 2.9 | 0.1157 | Yes |
| NOS2 | 1139 | 2.801 | 0.1285 | Yes |
| IL6 | 1196 | 2.763 | 0.1417 | Yes |
| ITGAL | 1215 | 2.743 | 0.1555 | Yes |
| CCL4 | 1276 | 2.679 | 0.1682 | Yes |
| CD40 | 1345 | 2.606 | 0.1803 | Yes |
| CCL2 | 1435 | 2.54 | 0.1918 | Yes |
| IL7 | 1742 | 2.391 | 0.1986 | Yes |
| CCL7 | 1938 | 2.348 | 0.2071 | Yes |
| KLRD1 | 1952 | 2.338 | 0.2189 | Yes |
| RPL9 | 2131 | 2.227 | 0.2271 | Yes |
| TAP1 | 2175 | 2.196 | 0.2377 | Yes |
| IRF8 | 2176 | 2.196 | 0.249 | Yes |
| RPS9 | 2177 | 2.195 | 0.2603 | Yes |
| IL27RA | 2397 | 2.077 | 0.267 | Yes |
| PF4 | 2728 | 1.905 | 0.2708 | Yes |
| IL11 | 2963 | 1.807 | 0.2759 | Yes |
| B2M | 3215 | 1.725 | 0.2802 | Yes |
| FAS | 3464 | 1.623 | 0.284 | Yes |
| RPS19 | 3479 | 1.618 | 0.2921 | Yes |
| CDKN2A | 3580 | 1.575 | 0.2984 | Yes |
| TAPBP | 3595 | 1.573 | 0.3063 | Yes |
| ST8SIA4 | 3622 | 1.56 | 0.3138 | Yes |
| IFNAR2 | 3693 | 1.537 | 0.3205 | Yes |
| CD28 | 3777 | 1.525 | 0.3268 | Yes |
| NCF4 | 3836 | 1.505 | 0.3335 | Yes |
| CD3G | 4122 | 1.42 | 0.3356 | Yes |
| CAPG | 4300 | 1.378 | 0.3395 | Yes |
| CCR1 | 4359 | 1.363 | 0.3455 | Yes |
| IL18 | 4518 | 1.315 | 0.3494 | Yes |
| RPL39 | 4844 | 1.224 | 0.3498 | Yes |
| RIPK2 | 4976 | 1.189 | 0.3535 | Yes |
| CFP | 5175 | 1.145 | 0.3558 | Yes |
| CD47 | 5297 | 1.117 | 0.3594 | Yes |
| HIF1A | 5529 | 1.073 | 0.3607 | Yes |
| GZMA | 5534 | 1.072 | 0.3662 | Yes |
| PSMB10 | 5743 | 1.023 | 0.3677 | Yes |
| CD3E | 5747 | 1.021 | 0.3729 | Yes |
| SIT1 | 5761 | 1.016 | 0.3779 | Yes |
| GZMB | 5823 | 1.014 | 0.382 | Yes |
| LCP2 | 6065 | 0.966 | 0.3826 | Yes |
| DYRK3 | 6066 | 0.965 | 0.3875 | Yes |
| CD86 | 6080 | 0.961 | 0.3922 | Yes |
| CRTAM | 6672 | 0.879 | 0.3861 | Yes |

**Tables. S10.** Top 50 enriched genes in Gene Set Enrichment Analysis for Interferon gamma pathway

| SYMBOL | RANK IN GENE LIST | RANK METRIC SCORE | RUNNING ES | CORE ENRICHMENT |
|---|---|---|---|---|
| VAMP5 | 27 | 4.729 | 0.0149 | Yes |
| PNP | 172 | 4.037 | 0.0254 | Yes |
| IL15 | 277 | 3.8 | 0.0359 | Yes |
| CCL5 | 364 | 3.681 | 0.0463 | Yes |
| CD69 | 412 | 3.594 | 0.0571 | Yes |
| IL6 | 419 | 3.588 | 0.0686 | Yes |
| PDE4B | 461 | 3.522 | 0.0793 | Yes |
| CMPK2 | 462 | 3.514 | 0.0908 | Yes |
| RBCK1 | 497 | 3.493 | 0.1015 | Yes |
| LYSMD2 | 540 | 3.447 | 0.1119 | Yes |
| UPP1 | 572 | 3.393 | 0.1224 | Yes |
| GPR18 | 656 | 3.257 | 0.1315 | Yes |
| CASP4 | 699 | 3.192 | 0.1411 | Yes |
| ST3GAL5 | 705 | 3.179 | 0.1513 | Yes |
| CASP1 | 749 | 3.132 | 0.1607 | Yes |
| IFI44 | 865 | 3.075 | 0.1686 | Yes |
| PLSCR1 | 908 | 3.049 | 0.1778 | Yes |
| APOL6 | 909 | 3.048 | 0.1877 | Yes |
| IDO1 | 993 | 2.967 | 0.1958 | Yes |
| IFI35 | 1014 | 2.945 | 0.205 | Yes |
| NFKBIA | 1057 | 2.897 | 0.2137 | Yes |
| BST2 | 1084 | 2.864 | 0.2225 | Yes |
| IFI27 | 1169 | 2.788 | 0.2301 | Yes |
| ISG20 | 1175 | 2.782 | 0.239 | Yes |
| CD40 | 1351 | 2.606 | 0.2443 | Yes |
| RNF31 | 1399 | 2.572 | 0.2518 | Yes |
| CCL2 | 1441 | 2.54 | 0.2593 | Yes |
| VAMP8 | 1444 | 2.537 | 0.2675 | Yes |
| ITGB7 | 1458 | 2.524 | 0.2755 | Yes |
| TXNIP | 1625 | 2.426 | 0.2804 | Yes |
| CD274 | 1646 | 2.414 | 0.2879 | Yes |
| METTL7B | 1651 | 2.412 | 0.2956 | Yes |
| IL7 | 1750 | 2.391 | 0.3016 | Yes |
| PSME2 | 1860 | 2.372 | 0.3073 | Yes |
| SELP | 1888 | 2.365 | 0.3145 | Yes |
| LAP3 | 1909 | 2.36 | 0.3218 | Yes |
| ZNFX1 | 1914 | 2.357 | 0.3294 | Yes |
| TNFAIP3 | 1920 | 2.355 | 0.337 | Yes |
| CCL7 | 1947 | 2.348 | 0.3442 | Yes |
| RIPK1 | 1986 | 2.332 | 0.351 | Yes |
| PTGS2 | 1996 | 2.319 | 0.3584 | Yes |
| CXCL10 | 2022 | 2.303 | 0.3654 | Yes |
| TOR1B | 2051 | 2.284 | 0.3724 | Yes |
| IRF9 | 2097 | 2.255 | 0.3789 | Yes |
| PSMB9 | 2124 | 2.245 | 0.3857 | Yes |
| NFKB1 | 2151 | 2.221 | 0.3924 | Yes |
| NAMPT | 2161 | 2.209 | 0.3995 | Yes |
| TAP1 | 2187 | 2.196 | 0.4061 | Yes |
| IRF8 | 2188 | 2.196 | 0.4133 | Yes |
| GBP4 | 2273 | 2.152 | 0.4187 | Yes |

**Tables. S11**. TOP 50 enriched genes in Gene Set Enrichment Analysis for Interferon alpha pathway

| SYMBOL | RANK IN GENE LIST | RANK METRIC SCORE | RUNNING ES | CORE ENRICHMENT |
|---|---|---|---|---|
| IL15 | 277 | 3.8 | 0.023 | Yes |
| CMPK2 | 462 | 3.514 | 0.0456 | Yes |
| CASP1 | 749 | 3.132 | 0.0635 | Yes |
| IFI44 | 865 | 3.075 | 0.0841 | Yes |
| PLSCR1 | 908 | 3.049 | 0.1059 | Yes |
| IFI35 | 1014 | 2.945 | 0.1257 | Yes |
| BST2 | 1084 | 2.864 | 0.1456 | Yes |
| IFI27 | 1169 | 2.788 | 0.1646 | Yes |
| ISG20 | 1175 | 2.782 | 0.1851 | Yes |
| RNF31 | 1399 | 2.572 | 0.2 | Yes |
| TXNIP | 1625 | 2.426 | 0.2138 | Yes |
| IL7 | 1750 | 2.391 | 0.2292 | Yes |
| PSME2 | 1860 | 2.372 | 0.2447 | Yes |
| LAP3 | 1909 | 2.36 | 0.2613 | Yes |
| CXCL10 | 2022 | 2.303 | 0.2762 | Yes |
| IRF9 | 2097 | 2.255 | 0.2915 | Yes |
| PSMB9 | 2124 | 2.245 | 0.3076 | Yes |
| TAP1 | 2187 | 2.196 | 0.3227 | Yes |
| GBP4 | 2273 | 2.152 | 0.337 | Yes |
| UBE2L6 | 2442 | 2.06 | 0.3492 | Yes |
| PSME1 | 2605 | 1.984 | 0.3609 | Yes |
| OGFR | 2614 | 1.978 | 0.3753 | Yes |
| LGALS3BP | 2710 | 1.929 | 0.3878 | Yes |
| IRF7 | 2723 | 1.923 | 0.4018 | Yes |
| HERC6 | 2736 | 1.906 | 0.4157 | Yes |
| DHX58 | 2743 | 1.905 | 0.4296 | Yes |
| LY6E | 2851 | 1.863 | 0.4414 | Yes |
| PSMB8 | 3059 | 1.788 | 0.4508 | Yes |
| MX1 | 3149 | 1.754 | 0.4622 | Yes |
| B2M | 3224 | 1.725 | 0.4736 | Yes |
| TRIM26 | 3329 | 1.684 | 0.4841 | Yes |
| TRIM21 | 3379 | 1.663 | 0.4955 | Yes |
| HELZ2 | 3410 | 1.655 | 0.5071 | Yes |
| UBA7 | 3500 | 1.611 | 0.5174 | Yes |
| CXCL11 | 3505 | 1.61 | 0.5292 | Yes |
| IRF1 | 3536 | 1.596 | 0.5404 | Yes |
| ADAR | 3550 | 1.587 | 0.5519 | Yes |
| BATF2 | 3567 | 1.585 | 0.5633 | Yes |
| NMI | 3569 | 1.585 | 0.575 | Yes |
| SLC25A28 | 3613 | 1.569 | 0.5858 | Yes |
| EPSTI1 | 3630 | 1.562 | 0.597 | Yes |
| STAT2 | 3671 | 1.547 | 0.6077 | Yes |
| SAMD9L | 3837 | 1.512 | 0.6159 | Yes |
| TDRD7 | 3886 | 1.502 | 0.6261 | Yes |
| PSMA3 | 3927 | 1.485 | 0.6363 | Yes |
| IFI30 | 4217 | 1.402 | 0.6414 | Yes |
| SP110 | 4221 | 1.398 | 0.6517 | Yes |
| USP18 | 4379 | 1.36 | 0.6589 | Yes |
| IFIT3 | 4761 | 1.25 | 0.6612 | Yes |
| PNPT1 | 4824 | 1.237 | 0.6692 | Yes |

| **Tables. S12.** Enriched genes in Gene Set Enrichment Analysis for cytosolic DNA sensing pathway | | | | |
|---|---|---|---|---|
| SYMBOL | RANK IN GENE LIST | RANK METRIC SCORE | RUNNING ES | CORE ENRICHMENT |
| CCL5 | 363 | 3.681 | 0.0453 | Yes |
| IL6 | 419 | 3.588 | 0.0949 | Yes |
| CCL4 | 456 | 3.529 | 0.144 | Yes |
| CASP1 | 747 | 3.132 | 0.1829 | Yes |
| IRF3 | 864 | 3.073 | 0.2241 | Yes |
| PYCARD | 919 | 3.041 | 0.266 | Yes |
| RIPK3 | 983 | 2.972 | 0.3068 | Yes |
| NFKBIA | 1057 | 2.897 | 0.3463 | Yes |
| POLR3H | 1459 | 2.523 | 0.3746 | Yes |
| IL18 | 1511 | 2.5 | 0.4089 | Yes |
| POLR3D | 1788 | 2.384 | 0.4375 | Yes |
| RIPK1 | 1985 | 2.332 | 0.4668 | Yes |
| CXCL10 | 2021 | 2.303 | 0.4987 | Yes |
| NFKB1 | 2150 | 2.221 | 0.5276 | Yes |
| DDX58 | 2582 | 1.997 | 0.548 | Yes |
| POLR3F | 2705 | 1.931 | 0.573 | Yes |
| IRF7 | 2723 | 1.923 | 0.5998 | Yes |
| IKBKG | 3301 | 1.698 | 0.6133 | Yes |
| TREX1 | 3332 | 1.682 | 0.6365 | Yes |
| ZBP1 | 3359 | 1.671 | 0.6595 | Yes |
| AIM2 | 3463 | 1.631 | 0.6807 | Yes |
| ADAR | 3550 | 1.587 | 0.7015 | Yes |
| IKBKB | 4116 | 1.428 | 0.7114 | Yes |
| NFKBIB | 4523 | 1.319 | 0.7227 | Yes |
| POLR3K | 4527 | 1.318 | 0.7412 | Yes |
| POLR3A | 4751 | 1.251 | 0.7548 | Yes |
| RELA | 4948 | 1.2 | 0.7682 | Yes |
| MAVS | 5569 | 1.065 | 0.772 | Yes |
| TBK1 | 5607 | 1.056 | 0.7862 | Yes |

**Table S13.** Antibody list

| Application | Target | Manufacturer |
|---|---|---|
| **Western Blot** | RAD51(8875, lot#5) | Cell Signaling Technology |
| | IRF-3(4302, lot#7) | |
| | p-IRF-3(Ser396)(29047, lot#4) | |
| | STING(13467, lot#6) | |
| | p-STING(S365)( 72971, lot#3) | |
| | Phosphor-Histone H2A.X(Ser139)(9718, | |

| | | |
|---|---|---|
| | lot#21) | |
| | β-tubulin(2146S) (lot #7) | |
| | GAPDH(5174)(lot #8) | |
| | Cyclophilin B(43603) (lot #6) | |
| | Anti-rabbit IgG, HRP-linked(7074) (lot #31) | |
| **Immunostaining** | Phosphor-Histone H2A.X(Ser139)(9718, lot#21) | Cell Signaling Technology |
| | Ki-67(IHC Formulated)(12202, lot#6) | Cell Signaling Technology |
| | RAD51(ab176458, lot#GR60659-35) | Abcam |
| | Fluorescein goat anti-rabbit IgG(H+L)(F2765, lot#2069794) | Invitrogen |
| | AF594 goat anti-rabbit IgG(H+L)(A32740, lot#UG288488) | Invitrogen |
| **Flow Cytometry** | CD45-PerCP(103130, Clone# 30-F11, Lot# B280746) | BD |
| | CD4-BUV737(612761, Clone# GK1.5, Lot#0058133) | BD |
| | CD8-BV480(566096, Clone# 53-6.7 (RUO), Lot# 1165100) | BD |
| | NK1.1-APC(550627, Clone#PK136, Lot#0170455) | Biolegend |
| | IFNγ -PE-Cy7 (505825, Clone# XMG1.2, Lot# 1243760) | Biolegend |
| | Granzyme B-Alexa Fluor 647(515405, Clone# GB11, Lot# B301363) | Biolegend |
| | CD24 -PE-Cy7(560536, Clone#M1/69, Lot#2301612) | BD |
| | pIRF3-Alexa Fluor 647(10327, Lot#4) | Cell Signaling Technology |
| | pTBK1-PE(13498, Lot#7) | Cell Signaling Technology |

## Reference


1 Sun, J. *et al.* High Loading of Hydrophobic and Hydrophilic Agents via Small Immunostimulatory Carrier for Enhanced Tumor Penetration and Combinational Therapy. *Theranostics* **10**, 1136-1150, doi:10.7150/thno.38287 (2020).

2 Wang, J., Wolf, R. M., Caldwell, J. W., Kollman, P. A. & Case, D. A. Development and testing of a general amber force field. *Journal of computational chemistry* **25**, 1157-1174, doi:10.1002/jcc.20035 (2004).

3 Gaussian 16 Rev. C.01 (Wallingford, CT, 2016).

4 Wang, J., Wang, W., Kollman, P. A. & Case, D. A. Automatic atom type and bond type perception in molecular mechanical calculations. *Journal of molecular graphics & modelling* **25**, 247-260, doi:10.1016/j.jmgm.2005.12.005 (2006).
5 Case, D. *et al. Amber 2018*. (2018).
6 Rocchia, W., Alexov, E. & Honig, B. Extending the Applicability of the Nonlinear Poisson−Boltzmann Equation: Multiple Dielectric Constants and Multivalent Ions. *The Journal of Physical Chemistry B* **105**, 6507-6514, doi:10.1021/jp010454y (2001).
7 Li, L. *et al.* DelPhi: a comprehensive suite for DelPhi software and associated resources. *BMC Biophysics* **5**, 9, doi:10.1186/2046-1682-5-9 (2012).
8 Wang, J., Hou, T. & Xu, X. Recent Advances in Free Energy Calculations with a Combination of Molecular Mechanics and Continuum Models. *Current Computer-Aided Drug Design* **2**, 287-306, doi:http://dx.doi.org/10.2174/157340906778226454 (2006).
9 Wang, E. *et al.* End-Point Binding Free Energy Calculation with MM/PBSA and MM/GBSA: Strategies and Applications in Drug Design. *Chemical Reviews* **119**, 9478-9508, doi:10.1021/acs.chemrev.9b00055 (2019).
10 Wang, J. & Hou, T. Develop and test a solvent accessible surface area-based model in conformational entropy calculations. *Journal of chemical information and modeling* **52**, 1199-1212, doi:10.1021/ci300064d (2012).
